# Virtual ChIP-seq: predicting transcription factor binding by learning from the transcriptome

**DOI:** 10.1186/s13059-022-02690-2

**Published:** 2022-06-10

**Authors:** Mehran Karimzadeh, Michael M. Hoffman

**Affiliations:** 1 Department of Medical Biophysics, University of Toronto, Toronto, ON Canada; 2 Princess Margaret Cancer Centre, Toronto, ON Canada; 3 Vector Institute, Toronto, ON Canada; 4 Department of Computer Science, University of Toronto, Toronto, ON Canada

## Abstract

Existing methods for computational prediction of transcription factor (TF) binding sites evaluate genomic regions with similarity to known TF sequence preferences. Most TF binding sites, however, do not resemble known TF sequence motifs, and many TFs are not sequence-specific. We developed Virtual ChIP-seq, which predicts binding of individual TFs in new cell types, integrating learned associations with gene expression and binding, TF binding sites from other cell types, and chromatin accessibility data in the new cell type. This approach outperforms methods that predict TF binding solely based on sequence preference, predicting binding for 36 TFs (MCC>0.3).

**Supplementary Information:**

The online version contains supplementary material available at (10.1186/s13059-022-02690-2).

## Background

Each TF can harmonize expression of many genes by binding to genomic regions that regulate transcription. Alteration in sequence or quantity of a given TF can be the primary cause of hereditary disorders, complex disease, autoimmune defects, and cancer [1].

TFs bind to accessible chromatin based on weak non-covalent interactions between amino acid residues and nucleic acids. DNA’s primary structure (sequence) [2], secondary structure (shape) [3], and tertiary structure (conformation) [4] all play roles in TF binding. Many TFs bind to DNA indirectly. In these cases, performance of models trained on in vitro data are poor when applied on in vivo experiments [5, 6]. To address this challenge, we must explore how to better model context-dependent TF binding.

Chromatin immunoprecipitation-sequencing (ChIP-seq) [7] can map the presence of a given TF in the genome of a biological sample. To map TFs, ChIP-seq requires a minimum of 1,000,000 to 100,000,000 cells, depending on properties of the TF itself and available antibodies. Large numbers of cells are not often available from clinical samples. Therefore, it is impossible to systematically assess TF binding in most disease systems. Assessing chromatin accessibility through transposase-accessible chromatin using sequencing (ATAC-seq) [8], however, requires only hundreds or thousands of cells. While chromatin accessibility does not determine TF binding exclusively, several methods use this information together with knowledge of TF sequence preference, genomic conservation, and other genomic features to predict TF binding [9–11].

We propose that using more accurate tools to predict TF binding will allow understanding the role of TF binding in more contexts.

Several methods use unsupervised approaches such as hierarchical mixture models [9] or hidden Markov models [10] to identify TF footprint using chromatin accessibility data. These approaches use sequence motif scores to attribute footprints to different TFs. Sequence motif scores, however, suffer from high false positives (FPs) and high high false negatives (FNs) (Additional file 1: Fig. S1). Variation in sequence specificity and cooperative binding of some TFs prevents these methods from accurately predicting binding of all TFs.

Most studies use different benchmarking approaches. For example, some methods [9, 12] only assess prediction on genomic regions that match the TF’s sequence motif. By excluding ChIP-seq peaks not matching the TF’s sequence motif from benchmarking, these methods underestimate FN peaks and overestimate prediction accuracy. Recently, the Encyclopedia of DNA Elements (ENCODE)-DREAM in vivo TF Binding Site Prediction Challenge (DREAM Challenge) introduced guidelines for assessing TF binding prediction [13]. They recommend reporting both area under the receiver operating characteristic curve (auROC), which assesses FN predictions and the area under the precision-recall curve (auPR), which also assesses FPs. As multi-threshold integration metrics [14], both auROC and auPR assess model performance of a scoring classifier independent of score threshold used. All scoring classifiers, by definition, depend on a threshold for stratifying the predictions. In addition to auPR and auROC, we also report the performance of our model on a predefined threshold using the Matthews correlation coefficient (MCC), which ranges between −1 and 1 (higher values indicate better performance). MCC takes into account all of true positives (TPs), FPs, true negatives (TNs), and FNs; therefore, it does not bias against FNs for imbalanced classifications. [15, 16].

### Virtual ChIP-seq

Here, we introduce Virtual ChIP-seq, a novel method for more accurate prediction of TF binding in new cell types. Virtual ChIP-seq predicts TF binding by learning from publicly available ChIP-seq experiments, genomic conservation, and the association of expression of all genes with TF binding. It does so by learning a novel representation of the effect of transcriptome on TF binding and integrating various epigenomic and genomic features using a supervised multi-layer perceptron.

Virtual ChIP-seq also accurately predicts the locations of some DNA-binding proteins without known sequence preference. This would be impossible for most existing methods, which rely on sequence preference. Strictly speaking, only some of these proteins are TFs. We use the term *chromatin factors* [17] in this paper to refer to factors subject to ChIP.

Virtual ChIP-seq predicted binding of 36 chromatin factors in new cell types with a minimum MCC of 0.3. Eight of these chromatin factors (GTF2F1, HCFC1, HDAC2, NRF1, RAD21, SIN3A, SMC3, and TAF1) do not have DNA-binding domains and therefore are not TFs according to Lambert et al. [18]. We predicted binding of these 36 chromatin factors on 33 Roadmap Epigenomics [19] cell types and provide these predictions as a track hub for community use (https://virchip.hoffmanlab.org).

## Results

### Model, performance, and benchmarking

#### Datasets

For training, our method requires ChIP-seq data of each chromatin factor in as many cell types as possible, with matched RNA-seq data from the same cell types. We used ChIP-seq data (from Cistrome DB [20] and ENCODE [21]) and RNA-seq data (from Cancer Cell Line Encyclopedia (CCLE) [22] and ENCODE [23]) to assess Virtual ChIP-seq’s binding predictions for 63 chromatin factors in new cell types.

In addition to benchmarking on our own held-out test cell types, we wanted to compare against the DREAM Challenge [13]. To do this, we also used their datasets, which include ChIP-seq data for 31 chromatin factors. The DREAM Challenge included ChIP-seq data for only 12 of these chromatin factors in completely held-out cell types. Completely holding out cell types better fits the real-world scenarios that require binding site prediction. Using the datasets we generated, we had matched data in enough cell types to train and validate models for 9 of these 12 chromatin factors (CTCF, E2F1, EGR1, FOXA1, GABPA, JUND, MAX, REST, and TAF1).

#### Learning from the transcriptome

Different cell types have distinct transcriptomic and epigenomic states [24]. We hypothesized that some gene expression changes would lead to consistent and observable changes in chromatin factor binding. As an extreme example, eliminating expression of a chromatin factor would eventually eliminate binding of that chromatin factor genome-wide. To account for both direct and indirect effects of the expression of regulatory genes, one must model the dependency of each chromatin factor binding site on expression of all genes [25]. To exploit this model, we identified all of the genes with significantly positive or negative correlation with chromatin factor binding at any given genomic bin.

**Fig. 1:**
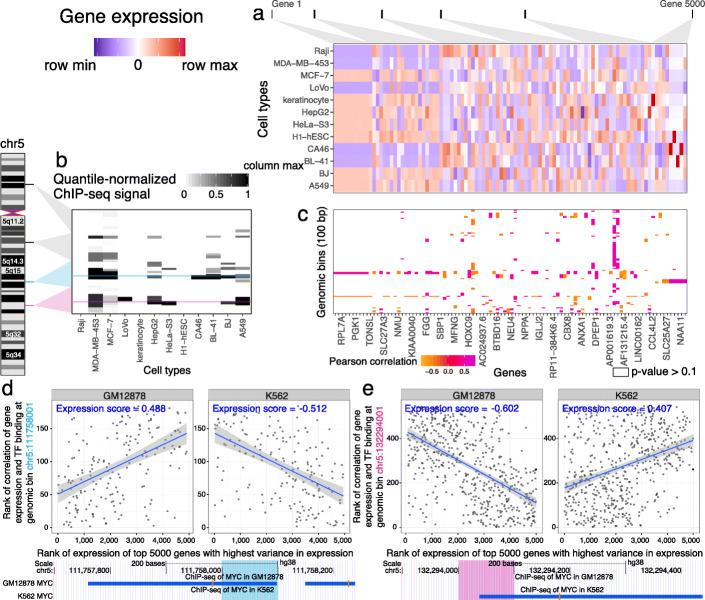
Virtual ChIP-seq learns from association of gene expression and chromatin factor binding at each genomic bin. This example shows Virtual ChIP-seq analysis for the MYC TF. **a** Gene expression levels for 5000 genes in 12 cell types. For simplicity of visualization, we showed only 100 of these genes in the matrix and labeled only one quarter of the genes. We ranked RNA-seq RPKM expression values within each cell type. This matrix shows a subset of 5000 high-variance genes, sorted by variance of each gene’s expression between cell types. Blue: row minimum; white: median expression; red: row maximum. **b** ChIP-seq signal for 100 bp bins in 12 cell types, taken from four larger regions (25 bins each) on chromosome 5. We quantile-normalized ChIP signal from MACS software among cell types. This matrix shows a subset of the 54,037 bins on chromosome 5 which have TF binding in at least one training cell type. White: column minimum (0.0); black: column maximum (1.0). Cyan: a region in the *NREP* locus with *MYC* binding in GM12878; magenta: a region upstream of *SLC22A4* with *MYC* binding in K562. **c** Association matrix: gene expression–ChIP signal correlation between 100 genomic bins and 5000 high-variance genes. This is a subset of the larger 54,037 ×5000 association matrix for chromosome 5. Each cell shows the Pearson correlation for 12 cell types between expression for a particular gene and ChIP signal at a particular genomic bin. Orange: negative correlation; white: p-value of Pearson correlation greater than 0.1 (NA); Purple: positive correlation. **d** (*Top*) Expression score plots for a 100 bp bin in the *NREP* locus. Each plot has one point for each of 184 genes with non-NA correlation values at that bin in the association matrix. Each point displays the rank of correlation value for that gene among one row of the association matrix against the rank of expression for that gene among 5000 high-variance genes in (*left*) GM12878 and (*right*) K562 cell types. The expression score at a bin for a cell type is Spearman’s rank correlation coefficient *ρ* between those two ranks. Blue line: best linear fit to data; grey region: 95% confidence interval of the fit. (*Bottom*) UCSC Genome Browser display of 550 bp around that region. Blue rectangle: *MYC* ChIP-seq peak in GM12878 or K562. Here, *MYC* binds only in GM12878. **e** Expression score plot and Genome Browser display for a 100 bp bin upstream of *SLC22A4*. Here, *MYC* binds only in K562

For each chromatin factor, we created an *association matrix* measuring correlation between expression of genes with variant expression among different cell types and binding of that chromatin factor in previously collected datasets (Fig. 1a–c). In this matrix, each value corresponds to the Pearson correlation between ChIP-seq binding of that chromatin factor at one genomic bin and the expression level of one gene. We used missing values when there was no significant association between gene expression and chromatin factor binding (unadjusted *p*>0.1).

**Fig. 2:**
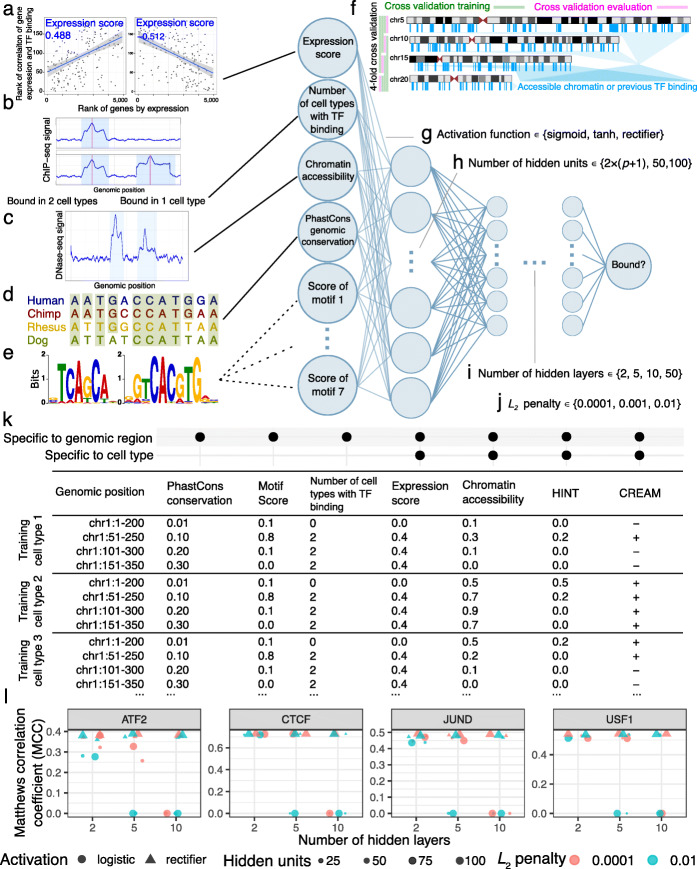
Optimizing and training a multi-layer perceptron. We used a number of features to predict chromatin factor binding in each bin. These include (a) expression score (Fig. 1d, e), (b) the number of training cell types with binding of that chromatin factor, (c) chromatin accessibility, (d) PhastCons genomic conservation in placental mammals, and (e) any sequence motif corresponding to that TF in the JASPAR database. In JASPAR, some chromatin factors have no sequence motifs, while others have up to seven different sequence motifs. This led to a number of features *p*∈ [ 4,11], excluding features from HINT footprints or CREAM peaks not used in the main model. (f) For each chromatin factor, we trained a multi-layer perceptron using these features for selected bins in four chromosomes (5, 10, 15, and 20). Specifically, we selected bins with accessible chromatin or ChIP-seq signal in at least one training cell type (selected regions with vertical blue bars are for illustration purpose). To optimize hyperparameters, we repeated the training process with different hyperparameters using fourfold cross validation, excluding one chromosome at a time. For each chromatin factor, we performed a grid search over (g) activation function (sigmoid, tanh, and rectifier), (h) number of hidden units per layer (2(*p*+1), 50, or 100), (i) number of hidden layers (2, 5, 10, or 50), and (j) *L*_2_ regularization penalty (0.0001, 0.001, or 0.01). We chose the quadruple of hyperparameters which resulted in the highest mean Matthews correlation coefficient (MCC) over all four chromosomes. (k) Schematic of the matrix of input features for training the multi-layer perceptron. We used a number of features to predict chromatin factor binding in each bin. (l) Cross-validation MCC of chr5 as a function of the number of hidden layers, for several other hyperparameter values. Size: number of hidden units within each layer. Shape indicates activation function: logistic (circle) or rectifier (triangle). Color indicates *L*_2_ regularization penalty: 0.01 (turquoise) or 0.0001 (orange)

We calculated an *expression score* for a chromatin factor in a new cell type using the association matrix and RNA-seq data for the new cell type, but no ChIP-seq data. The expression score is the Spearman correlation between the non-NA values for that genomic bin in the association matrix and the expression levels of those genes in the new cell type (Figs. 1d and 2a). We used the rank-based Spearman correlation to make the score robust against slight differences in analytical methodology used to estimate gene expression. We used the expression score as one of the variables in a multi-layer perceptron (see the “Methods” section).

The expression score can only provide predictions for genomic regions bound to the chromatin factor in training cell types. Across these genomic regions, expression score has a mean auPR of 0.16 (range 0–0.57). The expression score relies on the number of cell types used in calculating the correlation scores. Decreasing the number of training cell types resulted in decrease in auPR of the expression score in 12/17 TFs we tested. Only CTCF and EP300 had more than 8 training cell types. For both these factors, using fewer training cell types decreased prediction performance, a decrease only statistically significant in EP300 (Pearson correlation *r*=−0.42;*p*=0.003).

#### Learning from other predictive features

Virtual ChIP-seq includes as input for each genomic bin the frequency of the chromatin factor’s presence in existing ChIP-seq data (Fig. 2b). Since most chromatin factor binding occurs within accessible chromatin [26], we also used evidence of chromatin accessibility from DNase-seq or ATAC-seq (Fig. 2c).

While many intra-species genomic differences lie in the non-coding genome [27], we expect some regulatory elements to be conserved among closely related species. To learn from patterns of genomic conservation, we used PhastCons [28, 29] scores from a 7-way primate and placental mammal comparison (https://hgdownload.cse.ucsc.edu/goldenPath/hg38/phastCons7way) in our model (Fig. 2d).

We used sequence motif score where available (Fig. 2e; see the “Methods” section). For each TF, we represented sequence preference using the FIMO score of JASPAR sequence motifs of that TF or a similar TF. JASPAR has no motif for some chromatin factors, such as EP300. Where JASPAR has more than one motif for a TF, we included all of each TF’s motifs as features in its model (Additional file 2: Tables X1–X2). We used grid search to optimize the hyperparameters (Fig. 2f–j). Most changes of hyperparameter led to minimal differences in performance. Increasing the number of hidden units and hidden layers, however, particularly with a logistic activation function, inhibited the model from converging (Methods; Fig. 2l; Additional file 1: Fig. S4).

#### Virtual ChIP-seq predicts binding of 36 chromatin factors with high accuracy

We evaluated the performance of Virtual ChIP-seq in validation cell types (K562, PANC-1, MCF-7, IMR-90, H1-hESC, and primary liver cells) which we did not use their ChIP-seq data in calculating the expression score, training the multi-layer perceptron, or optimizing hyperparameters (Additional file 2: Tables X3–X4). Before predicting in new cell types, we chose a posterior probability cutoff for use in point metrics such as MCC, accuracy, and *F*_1_ score. When a chromatin factor had ChIP-seq data in more than one of the validation cell types, we chose the cutoff that maximizes MCC of that chromatin factor in H1-hESC cells. Then, we excluded H1-hESC when reporting threshold-requiring metrics. For these chromatin factors, we pre-set a posterior probability cutoff of 0.4, the mode of the cutoffs for other chromatin factors (Additional file 2: Table X5).

We evaluated the performance of Virtual ChIP-seq for 63 chromatin factors with binding in validation cell types (Additional file 2: Table X6; Additional file 1: Fig. S2–S3). For 36 of these chromatin factors, we achieved MCCs ranging from 0.31 to 0.73, *F*_1_ scores ranging from 0.21 to 0.73, and accuracy ranging from 0.99 to 1. For 59 of these chromatin factors, Virtual ChIP-seq predicted true chromatin factor binding in regions without conservation among placental mammals. For 44 out of 63 chromatin factors, Virtual ChIP-seq predicted true chromatin factor binding in regions without chromatin factor binding in any of the training ChIP-seq data. These novel predictions range from 4 in CTCF to 27,752 peaks in ATF2 (29.7% of total peaks, Additional file 2: Table X7). From these 63 chromatin factors, 43 are sequence-specific, and for all of these chromatin factors, Virtual ChIP-seq predicted true binding for regions that did not match the TF’s sequence motif. For 47 chromatin factors, Virtual ChIP-seq even correctly predicted chromatin factor binding in regions that did not overlap chromatin accessibility peaks (Additional file 2: Table X7). Most of these regions were frequently bound to the chromatin factor in publicly available ChIP-seq data. Along with an ablation study on removing individual features from training (Additional file 1: Fig. S4), and investigating the biological relevance of genes whose expression determined the expression score (Additional file 1: Fig. S5), these results showed that the multi-layer perceptron learned to leverage multiple kinds of information and predict chromatin factor binding accurately, even in the absence of features required by previous generations of binding site classifiers.

**Fig. 3:**
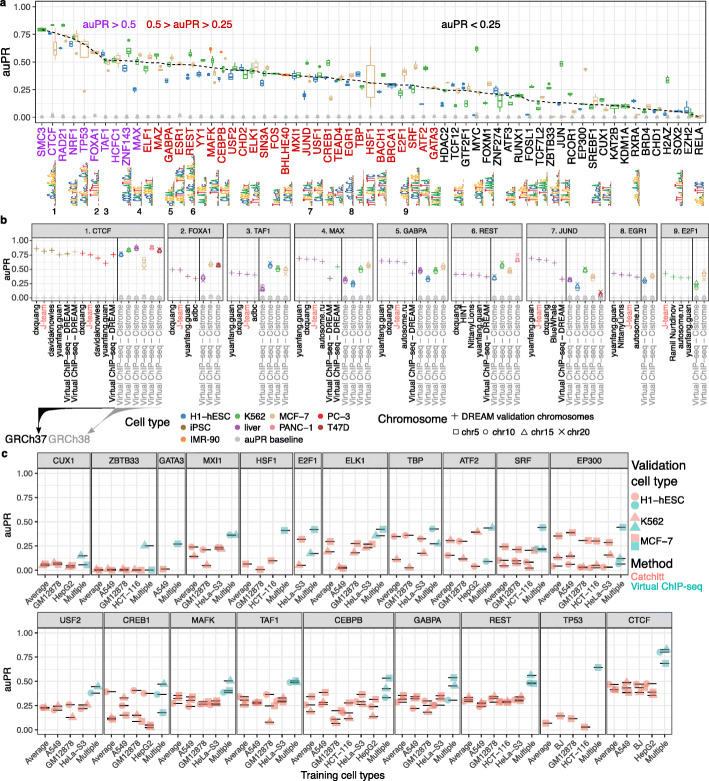
Virtual ChIP-seq predicts chromatin factor binding with high accuracy. Using ChIP-seq and RNA-seq data, we learned from the association of gene expression and chromatin factor binding for 63 chromatin factors. **a** Box plots show distribution of auPR among 4 chromosomes (5, 10, 15, and 20) for 63 chromatin factors assessed in four cell types (blue: H1-hESC; orange: IMR-90; green: K562; brown: MCF-7). Dashed line: medians. Grey shapes: prevalence of bound bins in the chromosome, the auPR baseline. Axis label colors categorize median auPR (purple: greater than 0.5, red: between 0.25 and 0.5, black: below 0.25). Sequence logos indicate one of a TF’s JASPAR motifs, when available. When multiple motifs existed, we displayed the shortest motif here. Numbers 1–9: The nine chromatin factors that the DREAM Challenge also evaluated in its final round. **b** We compared Virtual ChIP-seq’s performance to that of the top 4 performing methods in the DREAM Challenge across-cell type final round. For CTCF, MAX, GABPA, REST, and JUND, we had enough cell types to train and validate the performance of Virtual ChIP-seq on DREAM data. For these chromatin factors we trained on chromosomes 5, 10, 15, and 20 in training cell types and validated performance on merged data of chromosomes 1, 8, and 21 in validation cell types. For other chromatin factors, we trained the model and validated our performance using publicly available Cistrome and ENCODE data. auPR values are only directly comparable for the same cell type and test set. The black vertical line in each panel separates test sets based on genome assembly and source. Axis label color: reference genome assembly (black: GRCh37, grey: GRCh38). **c** We compared Virtual ChIP-seq’s performance to that of Catchitt, the co-winner of the ENCODE-DREAM Challenge on GRCh38 datasets. The *x*-axis shows the training cell type we used for training the model. Multiple: data of multiple cell types concatenated for training. Average: indicates average of posterior probability from models trained on each of the training cell types. We examined performance on three different validation cell types: H1-hESC (circle), K562 (triangle), and MCF-7 (rectangle). Turquoise: Virtual ChIP-seq. Orange: Catchitt. Black horizontal lines clarify the vertical position performance of each point

Virtual ChIP-seq predicts binding of 36 chromatin factors in validation cell types with MCC>0.3, auROC>0.86, and 0.19<auPR<0.84 (Fig. 3a; Table 1; Additional file 2: Table X6).

**Table 1:** Performance of Virtual ChIP-seq for 36 chromatin factors on validation cell types. Each row displays median values ± standard deviation of several performance metrics for prediction of a chromatin factor across 4 chromosomes for each available validation cell type. MCC, Matthews correlation coefficient; auROC, area under the receiver operating characteristic curve; auPR, area under the precision-recall curve; *N*, number of validation cell types for 36 chromatin factors with MCC>0.3. We reported auROC and auPR across all the validation cell types across all posterior probability cutoffs. Black chromatin factors: we found the posterior probability cutoff which maximized MCC in H1-hESC, and then reported *F*_1_, accuracy, and MCC of the other validation cell types

Chromatin factor	*F*_1_	Accuracy	MCC	auROC	auPR	*N*
ATF2	0.270 ±0.002	0.990 ±0.001	0.314 ±0.008	0.917 ±0.026	0.443 ±0.022	1
BHLHE40	0.334 ±0.021	0.997 ±0.000	0.356 ±0.010	0.974 ±0.002	0.382 ±0.010	1
CEBPB	0.510 ±0.091	0.992 ±0.002	0.515 ±0.072	0.964 ±0.017	0.534 ±0.073	3
CHD2	0.399 ±0.038	0.998 ±0.000	0.406 ±0.034	0.950 ±0.012	0.386 ±0.046	1
CREB1	0.362 ±0.131	0.997 ±0.002	0.371 ±0.121	0.868 ±0.135	0.335 ±0.174	2
CTCF	0.667 ±0.126	0.995 ±0.004	0.675 ±0.092	0.985 ±0.050	0.841 ±0.108	6
E2F1	0.256 ±0.097	0.998 ±0.002	0.314 ±0.078	0.978 ±0.019	0.291 ±0.105	2
ELF1	0.431 ±0.047	0.997 ±0.001	0.456 ±0.038	0.949 ±0.042	0.493 ±0.066	2
ELK1	0.430 ±0.069	1.000 ±0.000	0.465 ±0.054	0.991 ±0.009	0.420 ±0.054	2
ESR1	0.372 ±0.103	0.993 ±0.006	0.430 ±0.049	0.883 ±0.033	0.461 ±0.019	2
FOS	0.333 ±0.027	0.997 ±0.001	0.393 ±0.020	0.861 ±0.004	0.394 ±0.008	1
FOSL1	0.319 ±0.006	0.994 ±0.001	0.316 ±0.006	0.929 ±0.006	0.272 ±0.012	1
FOXA1	0.433 ±0.082	0.997 ±0.004	0.492 ±0.072	0.981 ±0.022	0.568 ±0.117	3
GABPA	0.298 ±0.049	0.994 ±0.002	0.393 ±0.036	0.986 ±0.012	0.496 ±0.036	3
GTF2F1	0.235 ±0.120	0.996 ±0.001	0.312 ±0.070	0.985 ±0.015	0.191 ±0.081	2
HCFC1	0.459 ±0.021	0.999 ±0.000	0.487 ±0.024	0.990 ±0.005	0.515 ±0.044	2
HDAC2	0.303 ±0.033	0.986 ±0.005	0.370 ±0.018	0.948 ±0.051	0.281 ±0.040	2
HSF1	0.350 ±0.149	1.000 ±0.000	0.378 ±0.145	0.999 ±0.012	0.309 ±0.240	1
JUN	0.218 ±0.127	0.998 ±0.001	0.311 ±0.153	0.983 ±0.009	0.456 ±0.257	2
JUND	0.341 ±0.163	0.993 ±0.002	0.386 ±0.135	0.979 ±0.019	0.326 ±0.161	4
MAFK	0.354 ±0.041	0.997 ±0.001	0.423 ±0.028	0.989 ±0.005	0.513 ±0.103	3
MAX	0.400 ±0.045	0.996 ±0.002	0.444 ±0.059	0.961 ±0.012	0.491 ±0.111	3
MAZ	0.370 ±0.025	0.997 ±0.001	0.422 ±0.019	0.987 ±0.005	0.493 ±0.070	2
MXI1	0.394 ±0.018	0.999 ±0.000	0.402 ±0.017	0.993 ±0.004	0.381 ±0.025	1
NRF1	0.658 ±0.042	1.000 ±0.000	0.664 ±0.038	0.994 ±0.014	0.720 ±0.051	3
RAD21	0.593 ±0.062	0.996 ±0.002	0.626 ±0.056	0.983 ±0.033	0.740 ±0.095	3
REST	0.482 ±0.120	0.999 ±0.001	0.493 ±0.091	0.985 ±0.008	0.567 ±0.095	3
SIN3A	0.389 ±0.048	0.998 ±0.002	0.394 ±0.029	0.966 ±0.004	0.411 ±0.037	3
SMC3	0.733 ±0.016	0.999 ±0.000	0.734 ±0.016	0.998 ±0.001	0.792 ±0.018	1
SRF	0.353 ±0.060	0.998 ±0.001	0.364 ±0.070	0.982 ±0.008	0.365 ±0.115	2
TAF1	0.378 ±0.073	0.999 ±0.001	0.437 ±0.097	0.987 ±0.009	0.490 ±0.168	3
TEAD4	0.344 ±0.061	0.990 ±0.002	0.385 ±0.020	0.967 ±0.023	0.343 ±0.019	2
TP53	0.275 ±0.103	1.000 ±0.000	0.382 ±0.086	1.000 ±0.008	0.660 ±0.222	1
USF1	0.353 ±0.047	0.993 ±0.001	0.382 ±0.040	0.891 ±0.012	0.372 ±0.046	1
USF2	0.410 ±0.040	0.999 ±0.000	0.427 ±0.028	0.982 ±0.007	0.437 ±0.032	1
YY1	0.397 ±0.049	0.996 ±0.001	0.408 ±0.058	0.945 ±0.043	0.417 ±0.104	2

#### Comparison with DREAM Challenge

DREAM Challenge rules forbid using genomic conservation or ChIP-seq data as training features directly. This also excludes the expression score, as creating its association matrix relies on ChIP-seq data. The challenge also required training and validation on its own provided datasets. These datasets have ChIP-seq data in only a few cell types. This restricts Virtual ChIP-seq’s approach which leverages all publicly available datasets. The DREAM Challenge ChIP-seq datasets use only two replicates for each experiment and requires that peaks have a irreproducibility discovery rate (IDR) [30] of less than 5%. In these cases, we included peaks that pass a false discovery rate (FDR) threshold of 10^−4^ in at least two replicates (Additional file 2: Table X8).

We trained and benchmarked Virtual ChIP-seq both on DREAM Challenge data, and on the Cistrome database, which provides a higher number of ChIP-seq datasets. The DREAM Challenge assessed participant entries by measuring performance on three validation chromosomes (chr1, chr8, and chr21), combined.

To assess performance of Virtual ChIP-seq on DREAM Challenge data, we did the same. To assess performance on Cistrome data, however, we measured performance on each chromosome independently. This allowed us to examine the variance in performance among these chromosomes.

Although Virtual ChIP-seq used features not allowed in the DREAM Challenge, comparing with DREAM Challenge participants is the only sound way to show how any method including these features compares to the state of the art. Leading DREAM Challenge methods potentially could improve their performance by including the features used by Virtual ChIP-seq. We compared Virtual ChIP-seq with DREAM Challenge results when we trained and validated on either Cistrome DB data or DREAM Challenge data.

#### Prediction accuracy varies by TF

The final submission round of the DREAM Challenge evaluates predictions for 12 chromatin factors in held-out cell types. The datasets we used, however, allow us to predict binding of 63 chromatin factors in new cell types. Of these chromatin factors, 41 are unique to our dataset and do not overlap any of the DREAM Challenge chromatin factors (Additional file 2: Table X9).

For CTCF, FOXA1, TAF1, and REST, Virtual ChIP-seq had a higher auPR in at least one validation cell type than any DREAM Challenge participant [31, 32]. For EGR1 and E2F1, Virtual ChIP-seq performed better than at least one of the four top-performing methods of the challenge in one of the validation cell types (Fig. 3b). For MAX, GABPA, and JUND, the 4 best-performing methods achieved a higher performance than Virtual ChIP-seq. DREAM Challenge and Cistrome ChIP-seq peak calls had different class imbalances, making auPR statistics not directly comparable (Additional file 2: Table X9). The differences in class prevalence are both minor and in diverging directions. Because of this, they do not bias the baseline auPR of evaluation on Cistrome datasets in a particular direction when compared to evaluation on DREAM Challenge datasets.

The power of Virtual ChIP-seq to learn from the transcriptome data diminishes when fewer cell types are available, as in the DREAM Challenge data. Nonetheless, when trained on DREAM Challenge data, Virtual ChIP-seq outperformed 13/14 DREAM Challenge participants when predicting CTCF binding in PC-3 cells. When predicting CTCF binding in induced pluripotent stem cell (iPSC) cells, Virtual ChIP-seq had a higher auPR than 8/14 Challenge participants. The Virtual ChIP-seq auPR for binding of REST in liver was also higher than that of 9/14 DREAM Challenge participants (Additional file 2: Table X9).

Virtual ChIP-seq predicted binding of 36 chromatin factors with a median MCC>0.3. These 36 chromatin factors had an auPR between 0.27 and 0.84 (Table 1). Some of these chromatin factors show high levels of consistent binding among different cell types, which makes predictions easier. The fraction of bins bound to a chromatin factor in at least half of training cell types, however, varies between 0 and 15.75% across all chromatin factors. For some chromatin factors, Virtual ChIP-seq fails to predict binding accurately (auPR<0.3). Chromatin factors with low auPR and low MCC include chromatin modifiers such as KAT2B, KDM1A, and EZH2 and chromatin binding proteins such as CHD1 and BRD4. Chromatin factors with low prediction accuracy include ATF2, CUX1, E2F1, EP300, FOSL1, FOXM1, JUN, RCOR1, RELA, RXRA, SREBF1, TCF12, TCF7L2, and ZBTB33.

#### Comparison with Catchitt

J-Team tied for the first place in the ENCODE-DREAM Challenge with Yuanfang Guan’s team. On DREAM Challenge datasets, Virtual ChIP-seq only outperformed Yuanfang Guan when predicting CTCF binding in iPSC and PC-3 and only outperformed Catchitt [32] when predicting CTCF binding in PC-3 and REST in liver. In addition to the specific nature of CTCF as a chromatin factor, we hypothesized that the higher number of training cell types for CTCF compared to other chromatin factors contributed to the superior performance of Virtual ChIP-seq. To test this, we trained Catchitt on the same training cell types and datasets as Virtual ChIP-seq (see the “Methods” section). In addition to sequence motifs available for the chromatin factor in multiple databases, we used 44 sequence motif features from Epigram and DNase-seq peaks provided by the authors (Additional file 2: Table X10). Virtual ChIP-seq outperformed Catchitt for all of the 20 chromatin factors we used for the comparison, excluding CREB1, EP300, and ATF2 (Fig. 3c).

J-Team released their simplified algorithm as *Catchitt* and indicated that Catchitt performs almost as well as their original implementation. To investigate whether Catchitt can outperform Virtual ChIP-seq if trained on the same GRCh38/hg38 labels, we re-trained Catchitt. Since Catchitt requires a specific format of labels for training, we also used IDR-thresholded narrowPeak files and used the Catchitt *label* module to generate peak labels. We trained Catchitt on those datasets and evaluated performance of Catchitt as well as Virtual ChIP-seq on those label sets. Although we disadvantaged Virtual ChIP-seq by not re-training it on labels that Catchitt generated, Virtual ChIP-seq still outperformed Catchitt.

We emphasize that, unlike Catchitt, Virtual ChIP-seq uses ChIP-seq data of the same TF in training cell types as a feature. This comparison primarily shows that using these additional features, some of which we proposed and used here for the first time, makes Virtual ChIP-seq a superior method. We suggest that using these features will boost the performance of all models, including future models derived from Catchitt.

#### Comparison with Avocado

While the participants of the ENCODE-DREAM challenge did not use any ChIP-seq data for training, other methods, such as Avocado [33], do use existing ChIP-seq data for training. Avocado uses ChIP-seq, DNase-seq, ATAC-seq, and RNA-seq data to impute epigenomic and transcriptomic signals over 25 bp genomic bins.

To benchmark with another method which used ChIP-seq data during training, we compared Virtual ChIP-seq predictions in 32 chromatin factors across 3 cell types with Avocado imputations. Specifically, we compared the predictions of Virtual ChIP-seq in 200 bp genomic windows with both mean and maximum of Avocado imputations over those windows.

For most of the examined chromatin factors, Virtual ChIP-seq consistently had higher predictive performance than Avocado. Specifically, in 21 of the chromatin factors, Virtual ChIP-seq consistently had higher auPR (median: 0.51) than Avocado (median: 0.33; Additional file 1: Fig. S6). For RXRA, ATF3, and GTF2F1, Avocado had higher auPR (median: 0.32) than Virtual ChIP-seq (median: 0.18) in predicting among all of 3 cell types (Additional file 1: Fig. S6). For the remaining 8/32 chromatin factors, however, neither method consistently had the best auPR in all cell types (Additional file 1: Fig. S6; Additional file 2: Table X13).

### A compendium of chromatin factor binding predictions for 33 tissues and cell types

#### Predicting chromatin factor binding in Roadmap datasets

The Roadmap Epigenomics Project [19] performed DNase-seq on 55 and RNA-seq on 39 human tissues and cell types, but not ChIP-seq of any chromatin factor. For 33 of these tissues, they produced matched DNase-seq and RNA-seq data. This makes the Roadmap data an ideal application for Virtual ChIP-seq.

**Fig. 4:**
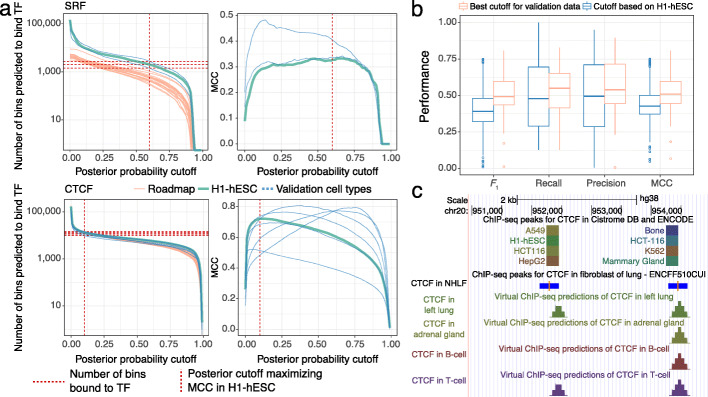
Chromatin factor binding predictions in validation cell types and Roadmap datasets. **a** Number of genomic bins that chromatin factor is predicted to bind (left) and MCC (right) as a function of posterior probability cutoff for SRF (top) and CTCF (bottom). This relationship is shown for H1-hESC (turquoise), 2 validation cell types for SRF (blue), and 6 validation cell types for CTCF (blue). Each curve represents predictions for one of the 4 chromosomes (chr5, chr10, chr15, and chr20). Left panels also show how many genomic bins are predicted to bind the chromatin factor in 18 Roadmap datasets (red). Vertical red dashed line: posterior probability cutoff which maximized MCC of the chromatin factor in H1-hESC. Horizontal red dashed lines: number of genomic bins with chromatin factor binding in validation cell types. **b** Boxplot of various performance measures when using the best cutoff for each dataset (red) and the optimal cutoff in H1-hESC (turquoise). **c** UCSC Genome Browser display of a 4000 bp region on chromosome 20 using the Virtual ChIP-seq track hub (https://virchip.hoffmanlab.org). The track hub has a supertrack for each chromatin factor. Each supertrack contains 34 tracks: one track specifying genomic bins bound by that chromatin factor in Cistrome and ENCODE, and one track for each of the 33 Roadmap cell types with predictions for that chromatin factor. This example shows parts of the track hub related to CTCF, including a track with experimental results in Cistrome DB and ENCODE with 7 out of 144 cell types enabled, and Virtual ChIP-seq predictions in left lung, adrenal gland, B-cell, and T-cell. The height of predictions indicates the number of overlapping genomic bins predicted to bind the chromatin factor, ranging between 0 and 4. Between are MACS2 narrow peak calls for CTCF in NHLFs from ENCODE (ENCFF510CUI). Blue: peaks; orange: peak summits

We generated an annotation similar to peak calls by converting the multi-layer perceptron’s posterior probabilities to a presence or absence call. The number of binding sites we predicted in other validation cell types and Roadmap data is similar to ChIP-seq peaks in other validation cell types (Fig. 4a).

Using the cutoff which maximized MCC in H1-hESC only slightly decreased performance measurements from what one could achieve with the optimal cutoff for each cell type (Fig. 4b). For example, the MCC score showed a median decrease of 0.06 and *F*_1_ score showed a median decrease of 0.1. This also affected precision (fraction of correct predictions compared to all positive predictions) to a lesser extent than recall (fraction of correct predictions compared to all positive labels).

As a community resource, we created a public track hub (https://virchip.hoffmanlab.org) with predictions for 33 Roadmap cell types (Fig. 4c). This track hub contains predictions for 36 chromatin factors which had a median MCC>0.3 in validation cell types (Table 1).

## Discussion

Performing functional genomics assays to assess binding of all chromatin factors may never be possible in patient tissues. Scanning the genome for occurrences of each sequence motif results in a range of 200–2000 predictions/Mbp. In some cases, this is 1000 times more frequent than experimental data from ChIP-seq peaks. Similar observations led to a *futility conjecture* that almost all TF binding sites predicted in this way will have no functional role [34].

Nevertheless, there is more to TF binding than sequence preference. Most chromatin factors do not have any sequence preference [6] (Additional file 1: Fig. S1), and indirect TF binding through complexes of chromatin-binding proteins complicates predictions based solely on sequence specificity. In addition to the high number of FP motif occurrences, many ChIP-seq peaks lack the TF’s sequence motif. Therefore, relying on sequence specificity alone not only generates too many FPs, but also many FNs. We call this latter observation the *dual futility conjecture*, although it differs in degree from the original. To provide cell-type–specific predictions, we need to teach the model about cellular state by adding cell-type–specific features. This allows us to move beyond both futility conjectures.

ChIP-seq–based assays may still not properly reflect in vivo chromatin factor binding due to technical difficulties such as non-specific or low affinity antibodies [35] or false detection of unrelated factors in hyper-ChIPable regions [36]. More robust approaches in assessment of chromatin factor binding—such as CRISPR epitope tagging CRISPR epitope tagging ChIP-seq (CETCh-seq) [37], which does not rely on specific antibodies—may provide less noisy reference data for learning and prediction of chromatin factor binding.

Deep learning models can learn from transcriptome datasets [38]. By integrating the transcriptome with other epigenomic and genomic features, Virtual ChIP-seq predicted binding of 36 chromatin factors in new cell types, using from the new cell types only chromatin accessibility and transcriptome data. By learning from direct evidence of chromatin factor binding and the association of the transcriptome with chromatin factor binding at each genomic region, most use of sequence motif scores becomes redundant. As more ChIP-seq data in diverse cell types and tissues becomes available, our approach allows predicting binding of more chromatin factors with high accuracy.

## Methods

### Data used for prediction

#### Overlapping genomic bins

To generate the input matrix for training and validation, we used 200 bp genomic bins with sliding 50 bp windows. We excluded any genomic bin which overlaps with ENCODE blacklist regions (https://www.encodeproject.org/files/ENCFF419RSJ/@@download/ENCFF419RSJ.bed.gz). Except where otherwise specified, we used the Genome Reference Consortium GRCh38/hg38 assembly [39].

#### Chromatin accessibility

We used Cistrome DB ATAC-seq and DNase-seq narrowPeak files for assessing chromatin accessibility (Additional file 2: Table X8). We mapped the signal value of peak summits to all the bins overlapping that summit. In rare cases where a genomic bin overlaps more than one summit, we used the signal value of the summit closest to the p terminus of the chromosome When data were available from multiple experiments, we averaged signal values. Because Cistrome DB does not include raw data that one can use for DNase footprinting, we limited the analysis of Hidden Markov model-based Identification of Transcription factor footprints (HINT) TF footprinting and CREAM regulatory element clustering [40] to ENCODE DNase-seq experiments on GM12878, HCT-116, HeLa-S3, LNCaP, and HepG2.

#### Genomic conservation

We used GRCh38 primate and placental mammal 7-way PhastCons genomic conservation [28, 29] scores from the UCSC Genome Browser [41] (https://hgdownload.cse.ucsc.edu/goldenPath/hg38/phastCons7way). We assigned each bin the mean PhastCons score of the nucleotides within.

#### Sequence motif score

We used FIMO [42] (version 4.11.2) to search for motifs from JASPAR 2016 [43] to identify binding sites of each TF that have the sequence motif of that TF. We used the curated, non-redundant JASPAR database of vertebrate sequence motifs to avoid the unnecessary complexity of having similar redundant motifs. To get a liberal set of motif matches, we used a liberal *p*-value threshold of 0.001 and did not adjust for multiple testing. If the motif for the TF did not exist in JASPAR, we used other motifs with same initial 3 letters and counted any TF binding site which had overlap with any of those motifs. For example, SIX5 does not have a sequence motif in JASPAR. In that case, we used the sequence motif of SIX3 instead (Additional file 2: Table X1).

We also used FIMO and JASPAR 2016 to identify the sequence specificity of chromatin accessible regions. For this analysis, we used a FDR threshold of 0.01%. We used any sequence motif matching the initial 3 letters of a TF as a predictive feature of binding for that TF. For many chromatin factors, more than one motif matched this criterion, and we used all as independent features in the model (Additional file 2: Table X2). We used the average FIMO score of each motif present in each 200 bp. When the chromatin factor matched more than one motif, we used each one as an additional independent feature in the model, up to 7 motifs maximum.

#### ChIP-seq data

We used Cistrome DB and ENCODE ChIP-seq narrowPeak files. We only used peaks with FDR<10^−4^. When multiple replicates of the same experiment existed, we only considered peaks that passed the FDR threshold in at least two replicates. We considered bound only those genomic bins overlapping peak summits. We calculated prevalence of bound bins in each chromosome as

$$\text{prevalence} = \frac{\text{bound}}{\text{bound} + \text{unbound}} $$

and used it as an auPR baseline [44].

#### RNA-seq data

We downloaded an ENCODE expression matrix (https://public-docs.crg.es/rguigo/encode/ expressionMatrices/H.sapiens/hg19/2014_10/gencodev19_genes_with_RPKM_and_npIDR_oct2014.txt.gz) [23] with RNA-seq data for each gene, measured in reads per kilobase per million mapped reads (RPKM). We retrieved similar CCLE RNA-seq data using PharmacoGx [45]. Since these data are processed differently, we limited our analysis to Ensembl gene IDs shared between the two datasets, and ranked gene expression values by cell type. The two datasets have 4 shared cell types: A549, HepG2, K562, and MCF-7. Within each of these cell types, we examined the concordance of RNA-seq data between ENCODE and CCLE after possible transformations. The concordance correlation coefficient [46] of rank of RPKM (0.827) was higher compared to untransformed RPKM (0.007) or quantile-normalized RPKM (0.006; Welch t-test *p*=10^−6^). The DREAM Challenge, however, had processed RNA-seq of all cell types uniformly, allowing us to directly use transcripts per million reads (TPM) in analysis of DREAM Challenge datasets.

#### Expression score

We created an expression matrix for each chromatin factor with matched ChIP-seq and RNA-seq data in *N*≥5 training cell types with the following procedure:

1. We divided the genome into *M* 100 bp non-overlapping genomic bins.
2. We created a non-negative ChIP-seq matrix $\boldsymbol {C} \in \mathbb {R}_{\geq 0}^{M \times N}$ (Fig. 1a). We used signal mean among replicate narrowPeak files generated by MACS2 [47] for each of *M* bins and *N* cell types and quantile-normalized this matrix.
3. We row-normalized ***C*** to ***C***^***′***^, scaling the values of each row between 0 and 1.
4. We identified the *G*=5000 genes with the highest variance among the *N* cell types.
5. We created an expression matrix $\mathbf {E} \in \mathbb {R}_{\in [0,1]}^{N \times G}$ containing the row-normalized rank of expression each of the *G*=5000 genes in *N* cell types (Fig. 1b).
6. For each bin *i*∈ [ 1,*M*] and each gene *g*∈ [ 1,*G*], we calculated the Pearson correlation coefficient *A*_*i,g*_ between the ChIP-seq data for that bin ***C***^***′***^_*i*,:_ and the expression ranks for that gene ***E***_:,*j*_ over all cell types. If the Pearson correlation was not significant (*p*>0.1), we set *A*_*i,g*_ to NA. These coefficients constitute an association matrix $\boldsymbol {A} \in {(\mathbb {R}_{\in [-1,1]} \cup \{\text {{NA}}\})}^{M \times G}$ (Fig. 1c).

We performed power analysis of the Pearson correlation test using the R pwr package [48].

Power analysis identified which correlations the *p*>0.1 cutoff would exclude depending on the number of available cell types with matched ChIP-seq and RNA-seq data. For CTCF, which had the largest number of cell types available—21 cell types with matched ChIP-seq and RNA-seq—this cutoff provided 80% power to detect an absolute value of Pearson correlation |*r*|≥0.52. Many chromatin factors had only 5 cell types with matched data and the cutoff provided 80% power to detect only larger correlations, |*r*|≥0.92.

To predict ChIP-seq binding for a new cell type (Fig. 1d), we calculated an expression score for each genomic bin in that cell type. The expression score is Spearman’s *ρ* for expression of the same *G*=5000 genes in the new cell type with every row of the association matrix ***A***. Each of these rows represents a single genomic bin. An expression score close to 1 indicates that genes with high expression have high values in the association matrix, and genes with low expression genes have low values. An expression score close to −1 indicates that genes with high or low expression have opposite values in the association matrix (Fig. 1d).

### Training, optimization, and benchmarking

#### Selecting hyperparameters and training

We created an input matrix with rows corresponding to 200 bp genomic windows and columns representing the features described above. Specifically, these features included expression score (Fig. 2a), previous evidence of binding of chromatin factor of interest in publicly available ChIP-seq data (Fig. 2b), chromatin accessibility (Fig. 2c), genomic conservation (Fig. 2d), sequence motif scores (Fig. 2e), HINT footprints, and CREAM peaks. We used sliding genomic bins with 50 bp shifts, where most 200 bp bins overlap six other bins. This provided a maximum resolution of 50 bp in binding prediction. The result is a sparse matrix with 60,620,768 rows representing each bin in the GRCh38 genome assembly [39]. The sparse matrix used in the main model had between 4 and 11 columns, depending on the number of available sequence motifs.

We trained on an imbalanced subset of genomic regions which had chromatin factor binding or chromatin accessibility (FDR <10^−4^) in any of the training cell types. To speed the process of training and evaluation, we further limited training input data to four chromosomes (chr5, chr10, chr15, and chr20). For validation, however, we used data from these same four chromosomes in completely different cell types held out from training. We evaluated the performance on all of the 9,635,407 bins in these four chromosomes (Fig. 2f), not just those with prior evidence of chromatin factor binding or chromatin accessibility. To build a generalizable classifier that performs well on new cell types with only transcriptome and chromatin accessibility data, we concatenated input matrices from 12 training cell types: A549, GM12878, HepG2, HeLa-S3, HCT-116, BJ, Jurkat, NHEK, Raji, Ishikawa, LNCaP, and T47D (Additional file 2: Table X3).

#### The multi-layer perceptron

The multi-layer perceptron is a fully connected feed-forward artificial neural network [49]. Our multi-layer perceptron assumes binding at each genomic window is independent of upstream and downstream windows (Fig. 2). For each chromatin factor, we trained the multi-layer perceptron with adaptive momentum stochastic gradient descent [50] and a minibatch size of 200 samples. We used 4-fold cross validation to optimize hyperparameters including activation function (Fig. 2g), number of hidden units per layer (Fig. 2h), number of hidden layers (Fig. 2i), and *L*_2_ regularization penalty (Fig. 2j). For training, we only used genomic bins which overlapped chromatin accessibility peaks or previous evidence of chromatin factor binding in any of the training cell types. For assessing performance, however, we used all genomic bins of the chromosome. In each cross validation fold, we iteratively trained on 3 of the 4 chromosomes (5, 10, 15, and 20) at a time and assessed performance in the remaining chromosome. We selected the model with the highest average MCC [15] after 4-fold cross validation. MCC incorporates all four categories of a confusion matrix and assesses performance well even on imbalanced datasets [16]. For 23 chromatin factors, the optimal model had 10 hidden layers. For another set of 23 chromatin factors, the optimal model had 5 hidden layers. For the final 17 chromatin factors, the optimal model had only 2 hidden layers.

For 57 out of the 63 chromatin factors examined, the best-performing model had 100 hidden units in each layer—the maximum number of hidden units per layer examined in the grid search. For the remaining 6 chromatin factors, the optimal model had 10–24 hidden units in each layer. Different activation functions—hyperbolic tangent (tanh) or rectifier—proved optimal for different chromatin factors (Additional file 2: Table X4).

We investigated if chromatin factors with the same DNA binding domain (as reported in Lambert et al. [18]) also have similar optimized hyperparameters. All C2H2 zinc finger TFs (EGR1, CTCF, MAZ, REST, YY1, ZBTB33, ZNF143, and ZNF274) had a rectifier activation function, 100 hidden units, and *L*_2_ regularization penalty of 10^−4^. The number of hidden layers ranged from 2 to 10. The other DNA binding domains which had more than 4 TFs in our datasets, bHLH and bZIP, did not have the same hyperparameter among their TFs (Additional file 2: Table X4). There was also no significant correlation between number of hidden layers, hidden units, or activation function with performance of the model in validation cell types. Some models with higher numbers of hidden layers, particularly those with logistic activation function, failed to converge and resulted in cross-validation MCC of 0 (Fig. 2l).

#### Training and optimization

For the purpose of training and validating the model on Cistrome datasets, we only used chromosomes 5, 10, 15, and 20. These 4 chromosomes constitute 481.78 Mbp (15.6% of the genome). For training only, we excluded any genomic region without chromatin accessibility signal and previous evidence of chromatin factor binding. For validation and reporting performance, we included these regions, using the totality of the 4 chromosomes. We concatenated data from training cell types (A549, GM12878, HepG2, HeLa-S3, HCT-116, BJ, Jurkat, NHEK, Raji, Ishikawa, LNCaP, and T47D; Additional file 2: Table X3) into the training matrix.

We used Python 2.7.13, Scikit-learn 0.18.1 [51], NumPy 1.11.0, and Pandas 0.19.2 for processing data and training classifiers. We used the default Scikit-learn method [49] to initialize the multi-layer perceptron’s parameters *β* and coefficients *β*_0_. This uses random values from a uniform distribution. The support of the uniform distribution used depends on properties of the current layer *i* and the next layer *i*+1. Specifically, the maximum value *b* of the uniform distribution is a function of the number of the hidden units *u*_*i*_ in the current layer, the number of hidden units *u*_*i*+1_ in the next layer, and an activation factor *l* based on the activation function of the current layer. For sigmoid activation, *l*=2.0, and for other activation functions, *l*=6.0. For each layer *i*, Scikit-learn sets

$$b = \sqrt{\frac{l}{u_{i} + u_{i + 1}}} \text{.} $$

Scikit-learn samples each parameter *β*_*i*_ and each coefficient *β*_0,*i*_ from the uniform distribution $\mathcal {U}(-b, b)$.

#### Benchmarking

We used the R precrec package [52] to calculate auPR and auROC. Precision-recall (PR) curves better assess a binary classifier’s performance on imbalanced test data than receiver operating characteristic (ROC) [16, 44].

#### DREAM Challenge comparison

For comparison to DREAM results, we also trained and validated the Virtual ChIP-seq model on GRCh37 DREAM Challenge data. For training the model on DREAM Challenge datasets, we used the data of chr5, chr10, chr15, and chr20 of training cell types. We evaluated performance against the union of the DREAM validation chromosomes (chr1, chr8, and chr21) in validation cell types. For CTCF, we used MCF-7, PC-3, and iPSC for validation and trained on all other cell types (A549, H1-hESC, HeLa-S3, HepG2, IMR-90, and K562). For MAX, we used all cell types except liver and K562 for training. For GABPA, REST, and JUND, we used all cell types except liver for training. We compared these metrics to those of DREAM Challenge participants in the final round of cross–cell-type competition.

#### Comparison with Catchitt

We used the same set of sequence motifs as described in Keilwagen, Posch, and Grau [32]. Some of these sequence motifs, such as those derived from ChIP-seq peaks, are specific to each TF. Other sequence motifs, however, model epigenomic signatures such as chromatin accessibility and were used for all TFs.

We used the Catchitt software module *access* using GRCh38-aligned BAM files of each cell type to obtain the chromatin accessibility profile.

For Virtual ChIP-seq, we had generated B (bound) and U (unbound) labels. Catchitt, however, requires at least three labels: S (summit), B (non-summit but bound), and U (unbound). For comparison of Virtual ChIP-seq and Catchitt on GRCh38 datasets, we tried two different strategies. Once, we kept the B labels from our datasets at the edge of a peak region unchanged and swapped other B labels to S.

We also used the Catchitt software module *labels* to derive the four Catchitt labels A (ambiguous), S, B, and U from optimal and stringent IDR-thresholded narrowPeak files. When stringent IDR labels were not available for some ENCODE experiments, we used the optimal IDR labels for both the *c* and the *r* parameters as recommended by the authors.

For both set of labels, we trained Catchitt on the same training cell types and chromosomes as Virtual ChIP-seq. For evaluating the binary classification performance, we excluded ambiguous labels from the prediction space and considered either of S or B as TF binding.

We compared the performances of Virtual ChIP-seq and Catchitt using the auPR curve of predictions on chromosome 10 using identical labels in validation cell types.

### Clustering chromatin factors based on enrichment of their potential targets in GO terms

To identify groups of chromatin factors involved in similar biological processes, we examined the biological pathways that the targets of each chromatin factor regulate. We ranked genes according to the number of genomic regions where the expression of each gene correlated positively or negatively with chromatin factor signal. We calculated a correlation matrix for enrichment of targets of each chromatin factor in various biological processes. We performed hierarchical clustering on the correlation matrix. We sought to identify clusters of chromatin factors, and the best number of clusters between 2 and 10, inclusive. For use in this process, we created a Gaussian random matrix of 1681 rows and 113 columns as a control and calculated its correlation matrix. Then, we compared cluster stability between the original correlation matrix and the control for each potential number of clusters. To do this, we subsampled 75% of each correlation matrix rows twice without replacement. Then, we clustered chromatin factors in each matrix into the specified number clusters. For both of these clusterings, we constructed the set of every pair of chromatin factors present in the same cluster. We then calculated the Jaccard index between the first clustering’s constructed set and that of the second [53]. We repeated this subsampling and clustering process 50 times for each number of clusters. We picked the smallest number of clusters which had an increase in Jaccard index compared to the number of clusters one smaller only in the chromatin factor correlation matrix.

### Chromatin factor prediction on Roadmap data

We downloaded Roadmap DNase-seq and RNA-seq data aligned to GRCh38 from the ENCODE Data Coordination Center (DCC) [19]. For each DNase-seq narrowPeak file with matched RNA-seq, we predicted binding of 36 chromatin factors with MCC>0.3 in validation cell types (Table 1; Additional file 2: Table X6; https://virchip.hoffmanlab.org).

### Colors

For plots with three categories, we used a color palette optimized for viewers with deuteranopia (http://mkweb.bcgsc.ca/colorblind) and chose colors also distinguishable by those with protanopia and tritanopia.

For other plots, we either used the default ggplot2 [54] color palette or manually-adjusted ColorBrewer [55] palettes.

## Conclusions

Virtual ChIP-seq uses a fully connected neural network to integrate data from the transcriptome, chromatin accessibility, genomic context, and predict TF binding. Although Virtual ChIP-seq uses direct evidence of chromatin factor binding at each genomic region as one of the input features, it is able to correctly predict new peaks which do not exist in training cell types. Our datasets, using a combination of Cistrome DB and ENCODE, allow training and validating models for predicting binding in a more extensive 63 chromatin factors compared to DREAM Challenge datasets. Our provided predictions of binding of 36 high-confidence chromatin factors in 33 different Roadmap tissue types will allow the research community to better investigate epigenomics of disease affecting those tissues (https://virchip.hoffmanlab.org/). Our datasets should also accelerate the development of future machine learning methods by many groups.

## Supplementary Information

**Additional file 1** Portable Document Format (PDF): Supplemental text and figures [56–110].

**Additional file 2** Office Open XML Workbook (XLSX): Supplemental tables.

**Additional file 3** Review history.

## Data Availability

The datasets we used for training and validation are available at https://virchip.hoffmanlab.org. We have deposited in Zenodo the current version of our software [111], datasets [112], predictions for 36 TFs on Roadmap Epigenomics cell types [113], and predictions in Cistrome as well as the ENCODE-DREAM in vivo TF Binding Site Prediction Challenge [114]. We deposited the software on GitHub (https://github.com/hoffmangroup/virchip) and in the Bioconda package repository (https://anaconda.org/bioconda/virchip). We released the software under the GNU General Public License (GPL) version 3.0.
