# Supplementary material for: Virtual ChIP-seq: predicting transcription factor binding by learning from the transcriptome
Source: Genome Biol. 2022 Jun 10;23:126. doi: 10.1186/s13059-022-02690-2 (PMC9185870; doi:10.1186/s13059-022-02690-2)
Supplement: Supplementary file 1 — Additional file 1 Portable Document Format (PDF): Supplemental text and figures [56–110]. [file 13059_2022_2690_MOESM1_ESM.pdf]

Supplemental text and figures

For "Virtual ChIP-seq: predicting transcription factor binding by learning from the transcriptome".

Sequence motifs are absent in most TF binding sites

Most ChIP-seq peaks lack the TF's relevant sequence motif

Many computational tools predict transcription factor (TF) binding using sequence preference data [9, 10]. Most computational tools represent TF sequence preference in position weight matrix (PWM) format. PWMs encode the likelihood for presence of each nucleotide at different positions of a sequence motif. With tools such as FIMO [42], we can efficiently search and rank genomic regions that match TF sequence motifs as presented by PWMs.

One cannot determine a TF's binding sites based solely on its sequence preference. We can identify some additional properties, such as co-binding partners, from high-throughput experiments. For other properties, such as post-translational modifications to the TF, we lack corresponding large-scale data. Many post-translational modifications affect cellular localization, binding partners, and DNA-recognition of chromatin factors [56]. Therefore, we expect existing computational prediction methods to be more accurate for chromatin factors where context-specific variations such as post-translational modifications and co-binding partners contribute less to TF binding. For chromatin factors with more complex biology, however, we expect computational prediction methods to fail.

Using chromatin immunoprecipitation-sequencing (ChIP-seq) data on 201 chromatin factors in 54 different cell types, we investigated whether the majority of binding sites matched the sequence motif of the same TF. Among these 201 proteins, 76 lacked a sequence motif in JASPAR (Fig. S1a; Additional file 2: Table X1). Some of these motif-free proteins, such as EZH2 and HDAC, are chromatin-binding proteins rather than true TFs. For simplicity in describing the prediction task, we refer to them as chromatin factors. Others are TFs without known sequence preference. For sequence-specific TFs, the fraction of peaks that match a sequence motif ranges from 4.55% (for SIX5) to 94.2% (for CTCF) with a mean of 49.4% (Fig. S1b).

To investigate how the choice of p-value cutoff affected our findings, we explored different unadjusted p-value cutoffs for identifying a sequence motif match in a ChIP-seq peak. At a more stringent p-value cutoff of 10^{-6}, ChIP-seq peaks of no TF had more than 25% motif occupancy. Using a less stringent cutoff of 0.01 increased the motif occupancy of ChIP-seq peaks of most TFs to more than 75%. These results, however, differed little from having no statistical significance threshold at all (p-value ≤ 1; Fig. S1e). Accordingly, we chose the middle-ground p-value cutoff of 0.001 for our analysis (Fig. S1e).

To investigate the sensitivity of our observation to the JASPAR database, we replicated our motif analysis using HOCOMOCO (v11) [57]. The fraction of ChIP-seq peaks overlapping a sequence motif from HOCOMOCO and JASPAR databases correlate significantly (Pearson correlation $r = 0.9; p < 2 \times 10^{-16}$; Fig. S1e–f).

We hypothesized that ChIP-seq peaks without a strong motif for their chromatin factor might arise from ChIP capturing DNA bound by another protein interacting with the chromatin factor. To examine this hypothesis, we identified binding partners of chromatin factors from the STRING database (v11) [58] and looked for their sequence motifs. The ChIP-seq peaks of most chromatin factors (92/116) matched the sequence motif of one or more of interacting partners' sequence motifs. Often, peaks without the sequence motif of the chromatin factor, matched the sequence motif of the chromatin factor's interacting partners (first quartile: 5% of peaks, median: 25%, third quartile: 62%; Fig. S1f). This suggests that capture of binding interacting partners may prove responsible for the peaks from ChIP-seq of some chromatin factor that have no strong motif of that chromatin factor.

Many sequence motifs are not centrally enriched

Central enrichment measures how close a sequence motif occurs to a set of ChIP-seq peak summits. According to Bailey and Machanick [59], high central enrichment indicates direct TF binding. We used CentriMo [59] to measure central enrichment. We compared central enrichment between TFs with low motif occupancy (< 50% of ChIP-seq peaks contain the motif) and high motif occupancy (≥ 50% of peaks contain the motif; Fig. S1c). TFs with low motif occupancy had weaker central enrichment (t-test, $p = 0.02$; Fig. S1c–d). For example, 30.87% of ATF3 peaks overlapped with the MA0605.1 JASPAR motif. ATF3 peaks also had lower central enrichment than MAFK peaks, which had 74.29% overlap with the MA0496.1 JASPAR motif (Fig. S1d).

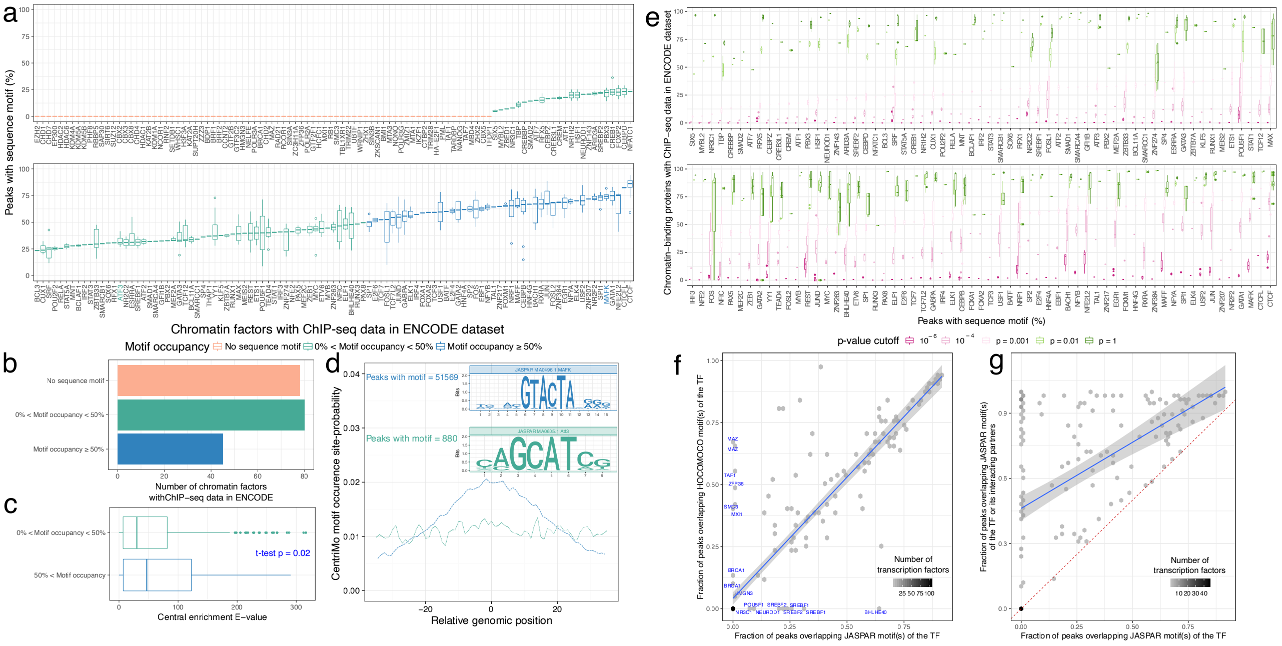

Fig. S1: **Most ChIP-seq peaks lack the TF's sequence motif.** **(a)** Fraction of Encyclopedia of DNA Elements (ENCODE) ChIP-seq peaks for a TF with any JASPAR sequence motif from the TF's family using the p-value threshold of 0.001. Boxplots show the distribution among datasets from different cell types and replicates. Horizontal line of boxplot: median. Box range: interquartile range (IQR). Whisker: most extreme value within quartile ±1.5 IQR. Individual points: outliers beyond a whisker. **(b)** Number of factors without a sequence motif in JASPAR (red), TFs where less than 50% of peaks have the sequence motif (low motif occupancy, green), and TFs where 50% or more of peaks have the sequence motif (high motif occupancy, blue). **(c)** Central enrichment [59] of a TF's motif is lower for TFs with motif occupancy of less than 50% compared to TFs with motif occupancy of 50% or more. **(d)** For TFs with a small number of peaks matching sequence motif of the same TF, such as ATF3, central enrichment of the motif is also low. In contrast, most MAFK peaks both contain its sequence motif and show central enrichment. **(e)** Similar to (a), for 5 different p-value thresholds (10^{-6}, 10^{-4}, 0.001, 0.01, and 1). **(f)** Mean fraction of each TF's peaks overlapping any sequence motif of the TF's family with a FIMO p-value cutoff of 0.01 from the HOCOMOCO database against the mean fraction from the JASPAR database. Blue text: TFs with a sequence motif in only one of the two databases. Blue line: linear regression best fit. Gray shadow: 5% confidence interval of the best fit. **(g)** Mean fraction of each TF's peaks overlapping any sequence motif of the TF's family against the mean fraction that overlap sequence motifs of either the TF's family or any of their interacting partners in the STRING database. Blue line: linear regression best fit. Gray shadow: 5% confidence interval of the best fit.

Exploring correct and false predictions

Features of true and false predictions

To better understand why the model sometimes predicted incorrectly, we examined predictions of 52 chromatin factors in validation chromosomes (chr5, chr10, chr15, and chr20) in K562. We investigated true positive (TP), false positive (FP), and false negative (FN) predictions. We excluded true negative (TN) predictions because their high numbers mainly reflect imbalanced class prevalence and potential ascertainment bias in the ground truth. Among the three labels, TP genomic bins varied from 0.19% for RELA to 58% for CTCF (Fig. S2a). For 24 of these 52 chromatin factors, most incorrect predictions were FN (Fig. S2a, left). For the other 28 chromatin factors, most incorrect predictions were FP (Fig. S2a, right).

We investigated presence and absence of predictive features among genomic bins labeled TP, FP, and FN. We defined presence of a feature as a positive value, and absence as a non-positive value. Expression score has values in $[-1, 1]$ when a region had chromatin factor binding in any of the training cell types that have matched RNA-seq data. For expression score, non-positive values include both 0 and negative values. All other input features only have values in $[0, 1]$. For most chromatin factors, the model performed better when all features were present. This means higher TP, lower FN, and lower FP (Fig. S2b).

For CTCF, incorrect predictions represented less than 5% of TPs when all predictive features were present, when only sequence motif was absent, or only the expression score was absent (Fig. S2b). Without presence of chromatin accessibility, the model made a higher number of false predictions, but still made some correct predictions.

The model only predicted novel binding sites not present in training cell types when the site matched the TF's sequence motif (Fig. S2b). For NRF1, MAFK, and ZNF274, the model made frequent FN predictions when expression score and sequence motif match were absent. REST, JUND, YY1, and E2F1 have more FP than FN. For these TFs, FP predictions were frequent when expression score and sequence motif match were absent. For ZBTB33, both FP and FN predictions were high when expression score and sequence motif match were both absent.

ZNF274 had only 117 correctly predicted binding sites and RELA had only 5 correctly predicted binding sites in the four validation chromosomes. In both of these cases, the model had low specificity and sensitivity, predicting a much higher number of FNs and FPs than TPs.

The expression score leverages similarity with training cell types to improve predictions

The expression score for a genomic bin is the Spearman correlation between expression of specific genes in a new cell type and a measure of how chromatin factor binding in that genomic bin correlates with expression of those genes among training cell types. For each genomic region, the expression score uses the expression values of a different set of genes to provide a low or high probability for chromatin factor binding in the new cell type.

We investigated whether the expression score serves as a way of encoding the ChIP-seq data of the training cell type with the most similar transcriptome to the new cell type. To do this, we randomly permuted expression scores across the genome. We identified bins that have TPs predictions with the original expression score but switch to FN with the permuted score. The correct predictions that require the original expression score usually had ChIP-seq peaks in one or more training cell type. In rare cases, these apparently expression-requiring predictions did not have corresponding binding in any of the training cell types. In these cases, the expression score may have contributed little to original prediction, but a permuted expression score penalized the bin below the prediction threshold.

We investigated the TF JUND in more detail. In JUND, 126 out of 1,155 expression-requiring TPs predictions did not exist in any of the training cell types (Fig. S3a, blue). Some of these true predictions (117/1,155) existed in only one of the training cell types (Fig. S3a, orange). We investigated correlation of the rank of expression of the top 5,000 genes with the highest variance among training cell types

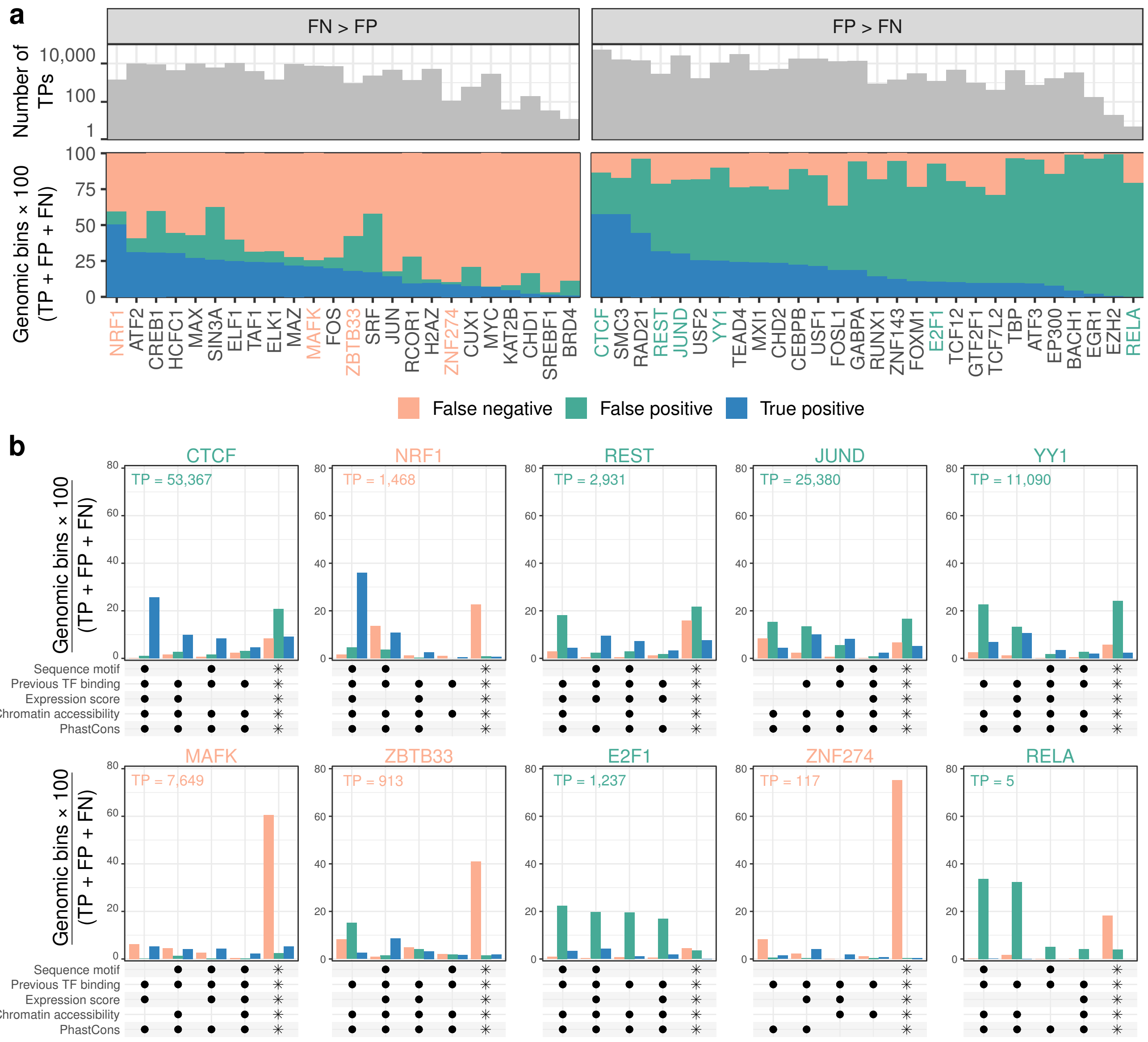

Fig. S2: **True and false predictions and associated features.** Fraction of potential chromatin factor binding sites in K562 categorized as FN (orange), FP (green), and TP (blue). This excludes any sites deemed TN. **(a)** Stacked bar plot of prediction categorization for the 52 chromatin factors with K562 ChIP-seq data, sorted by the fraction of TP genomic bins. Grey bars show number of TP predictions. (*Left*) 24 chromatin factors where FN fraction exceeded FP fraction. We selected 4 factors to examine in more detail below (orange names). (*Right*) 28 chromatin factors where FP fraction exceeded FN fraction. We selected 6 factors to examine in more detail below (green names). **(b)** UpSet [60] plot of prediction categorization in 10 factors given the 4 most common combinations of positive values for input features, and all other combinations (asterisks). Black dots indicate the features with positive values in each combination. Number of TPs indicated is in validation chromosomes (chr5, chr10, chr15, and chr20). We took the 10 factors from a wide range of those with best performance (top left) to worst performance, as sorted by ratio of TP to FP + FN.

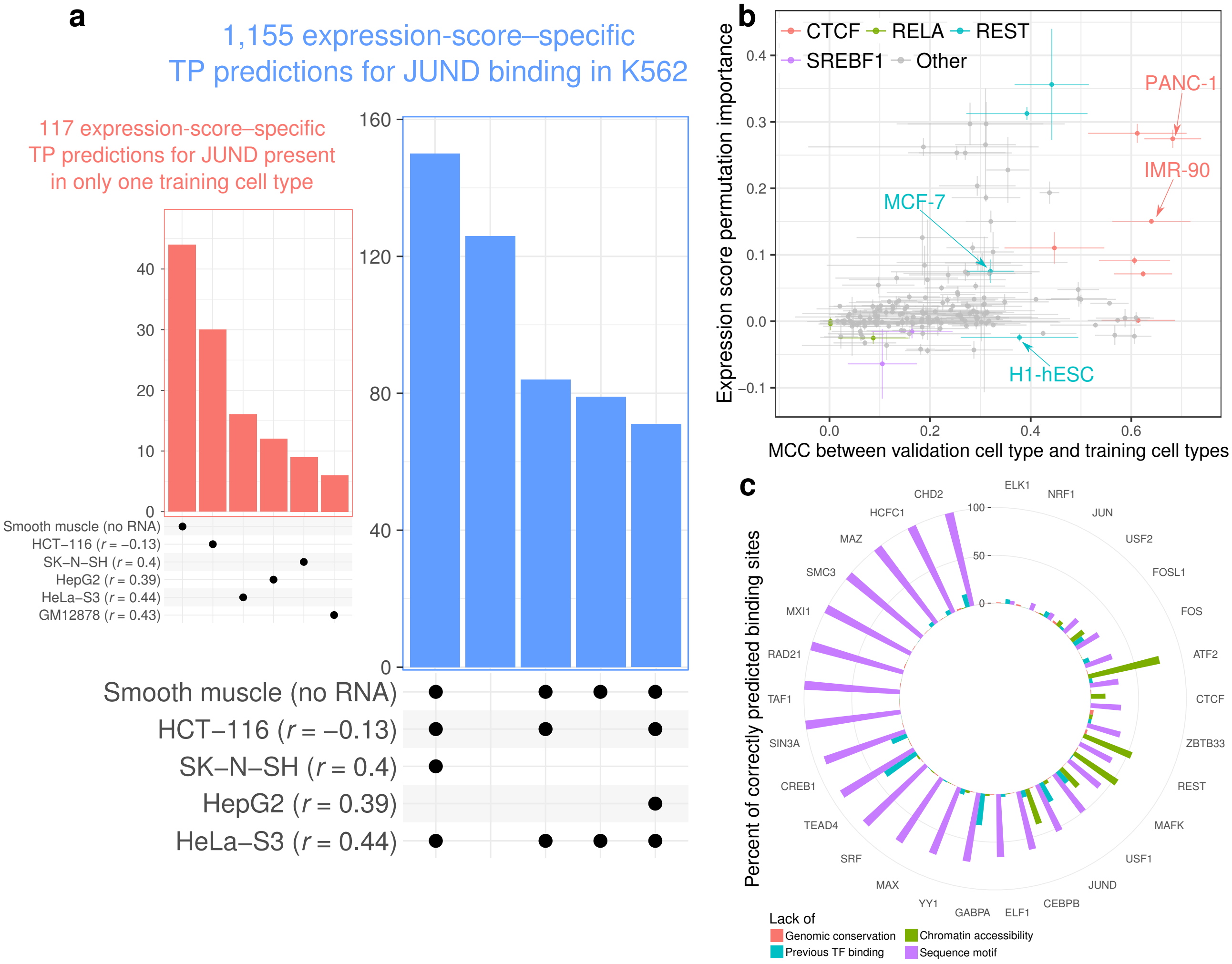

Fig. S3: **Expression score leverages similarity with training cell types.** **(a)** UpSet plots of TPs predictions of JUND binding in K562 which did not pass the posterior probability threshold when we permuted the expression score. Each bar represents a combination of training cell types with the binding site (black dots below plot). r: genome-wide correlation of rank of expression of the top 5,000 genes with highest variation among a training cell type with rank of expression of the same genes in K562. Smooth muscle lacked matched RNA-seq data. Blue plot: the top 5 combinations with the highest number of TPs genomic bins. Orange plot: the TPs predictions which were bound to chromatin factor in only one training cell type. **(b)** Scatter plot of expression score permutation importance for 160 pairs of 63 chromatin factors and 6 validation cell types against ChIP-seq peak similarity between that cell type and 1–10 training cell types. Permutation importance is the difference in area under the precision-recall curve (auPR) when permuting expression score. Similarity is measured by Matthews correlation coefficient (MCC) of validation cell type ChIP-seq peaks, treating each training cell type in turn as ground truth. Each point indicates median quantities, and error lines indicate median absolute deviation. **(c)** Bar plot of the fraction of binding sites for 29 chromatin factors correctly predicted on K562 validation chromosomes (chr5, 10, 15, and 20) which lacked particular predictive features. These features include genomic conservation (red), chromatin accessibility (green), sequence motif (turquoise), and evidence of chromatin factor binding in another cell type (purple). For chromatin factors with no sequence motif, we deemed every binding site to lack a sequence motif.

and the validation cell type K562 (Fig. S3a). The training cell type with the highest correlation was not necessarily the cell type with the highest number of expression-requiring predictions. For example, although the correlation among expression of all of the 5000 genes is highest between HeLa-S3 and K562 ($r = 0.44$), HCT-116 ($r = -0.13$) is the source of the highest number of correct expression score specific predictions. This is unsurprising since, for each region's expression score, we used only a subset of the 5,000 genes in the global calculation here. The other 912 predictions existed in 2 or more training cell types. This implies that, at least for JUND, the expression score did not simply encode ChIP-seq data of a single training cell type with the most similar global transcriptome to the new cell type.

We also examined whether the expression score's effectiveness depends on the similarity of chromatin factor binding among training and validation cell types. Under this hypothesis, we would expect high correlation between the expression score's contribution to model performance and the similarity of ChIP-seq data between the validation cell type and the training cell types. To examine this hypothesis, we calculated pairwise similarity in ChIP-seq data between the validation cell type and each training cell type. Due to the highly imbalanced class prevalence of ChIP-seq data, we used pairwise MCC as the similarity measure. We also calculated permutation importance [61], the difference in auPR when permuting the expression score ($\text{auPR} - \text{auPR}_{\text{permuted expression score}}$). Permutation importance indicates a feature's contribution to a predictive model.

For each validation cell type, we calculated the median MCC of its ChIP-seq data with that of training cell types and median expression score permutation importance among the 4 validation chromosomes (Fig. S3a). These two variables correlate in general (Spearman's $\rho = 0.41$; $p = 3 \times 10^{-8}$). CTCF binding in PANC-1 similarity with training cell types ranges from MCC $= 0.38$ to MCC $= 0.76$ (Fig. S3b). Only CTCF binding in IMR-90 has a higher similarity to training cell types (MCC $\in [0.35, 0.79]$). The permutation importance of CTCF predictions in PANC-1 is 0.27, while the permutation importance of CTCF predictions in IMR-90 is 0.15. The variation in correlation of similarity to training cell types and permutation importance of the expression score is more evident for REST (Fig. S3b). While the median similarity of REST binding with training cell types is 0.32 for MCF-7 and 0.38 for H1-hESC, the permutation importance for REST binding is 0.07 for MCF-7 but -0.02 for H1-hESC.

Using the expression score generally improved performance when validation cell types had similar TF location patterns to training cell types. For example, some validation cell types similar to the training cell types often had high expression score permutation importance (≥ 0.1) for CTCF (IMR-90, liver, MCF-7, PANC-1) and REST (K562, PANC-1). For RELA and SREBF1, however, all validation cell types had low expression score permutation importance (< 0.1), and low similarity of ChIP-seq data to training cell types (Fig. S3b).

Some correct predictions lack known predictive features

Many correctly predicted binding sites in K562 lack important predictive features of chromatin factor binding (Fig. S3c). Among 29 chromatin factors with MCC > 0.3 in K562, almost all correct predictions are in genomic bins conserved among placental mammals [28, 29]. The exceptions include 3.72% of predictions for ZBTB33, 2.11% of predictions for REST, 2.07% of predictions for USF2, 1.49% of predictions for NRF1, 1.47% of predictions for CHD2 and 0.18%–0.89% for other chromatin factors. Many correctly predicted binding sites for ATF2, MAFK, REST, CEBPB, USF1, FOSL1, and CTCF don't overlap chromatin accessibility peaks. We correctly predicted many binding sites for TEAD4, GABPA, JUND, CREB1, USF1, CHD2, and FOSL1 in regions which had no binding in training cell types. For all these factors except JUND, the nearest upstream or downstream genomic bin of these novel predictions in K562 bound the chromatin factor as well. The nearest training cell type binding site to these correct novel predictions were 50 bp–3.6 Mbp away. The nearest peak in training cell types for these novel predictions was not significantly closer compared to other K562 ChIP-seq peaks (Wilcoxon rank sum test; $p = 1$). In these cases, the multi-layer perceptron learned from other

available predictive features. For example, in TEAD4, all novel correctly predicted binding sites in validation chromosomes overlapped chromatin accessibility peaks. These correct predictions also had a mean PhastCons conservation of 0.182, significantly higher than the mean of 0.150 in other genomic bins (Welch t-test; $p < 2 \times 10^{-16}$).

The choice of input features determines prediction performance

The most important features

To evaluate the importance of each feature in our predictive model, we performed an ablation study on training data. First, we systematically removed features. Second, we fitted the model without these features on some of the training cell types (HeLa-S3, GM12878, HCT-116, LNCaP). Third, we evaluated performance on one held-out training cell type (HepG2; Additional file 2: Table X12). This ablation study did not use any of the validation cell types which we used for final evaluation of the model.

We called the effect of excluding an input feature substantive only when the average increase or decrease in auPR was at least 0.05. Excluding genomic conservation, sequence motif, HINT, or CREAM did not substantively change performance of the model for most chromatin factors (Fig. S4). Excluding chromatin accessibility, publicly available ChIP-seq data, and the expression score decreased performance in most chromatin factors. Excluding expression score substantively decreased median auPR in 13/21 chromatin factors, while excluding publicly available ChIP-seq data substantively decreased auPR in 18/21 chromatin factors.

To better understand the contribution of the expression score, we examined how expression score alone can predict chromatin factor binding at genomic regions bound by a factor in training cell types. Using expression score alone to predict EP300, a chromatin factor with training data in 16 cell types, resulted in low auPR (range: 0.01–0.02). In contrast, using expression score alone to predict USF2, a chromatin factor with training data in only 5 cell types, resulted in higher auPR (range: 0.26–0.45; Fig. S4d). These data show that properties of individual chromatin factors can have a larger role than number of training cell types in determining the expression score's predictive ability.

We examined further the relationship between number of training cell types and expression score accuracy by calculating the expression score for the same factor with different numbers of training cell types. CTCF had 23 training cell types, the largest number in our study. We calculated 19 expression score profiles for each of 5 to 23 training cell types. Each time, we randomly selected a subset of the

Fig. S4: *(Next page)*. **Virtual ChIP-seq's most important features consist in ChIP-seq data and expression score. (a)** Area under the Precision-recall curve (auPR) for predicting a chromatin factor's binding sites after training on only a subset of input features. We trained on five cell types (HeLa-S3, GM12878, HCT-116, and LNCaP) and predicted on either HepG2. Ablating a feature caused either substantive decrease (orange), substantive increase (turquoise), or no substantive change in auPR. An UpSet [60]-like matrix shows the subset of features used for each column. Dark grey strip above facet: when ablating Hidden Markov model-based Identification of Transcription factor footprints (HINT), CREAM, or sequence motifs substantively changed auPR. **(b)** Double-ended bar plot of the number of chromatin factors with average auPR increase or decrease of at least 0.05 when ablating each feature. Bars show the number of chromatin factors where ablation caused the average auPR to decrease (orange, left) or increase (turquoise, right). **(c)** Change in auPR for those chromatin factors with an average auPR increase or decrease of at least 0.05 when we excluded clusters of regulatory elements (CREAM), footprints (HINT), or sequence motifs. Backgrounds indicate auPR decrease (orange) or increase (turquoise). **(d)** auPR of expression score alone for predicting H1-hESC binding sites. Facets group chromatin factors with the same number of training cell types used to calculate the expression score.

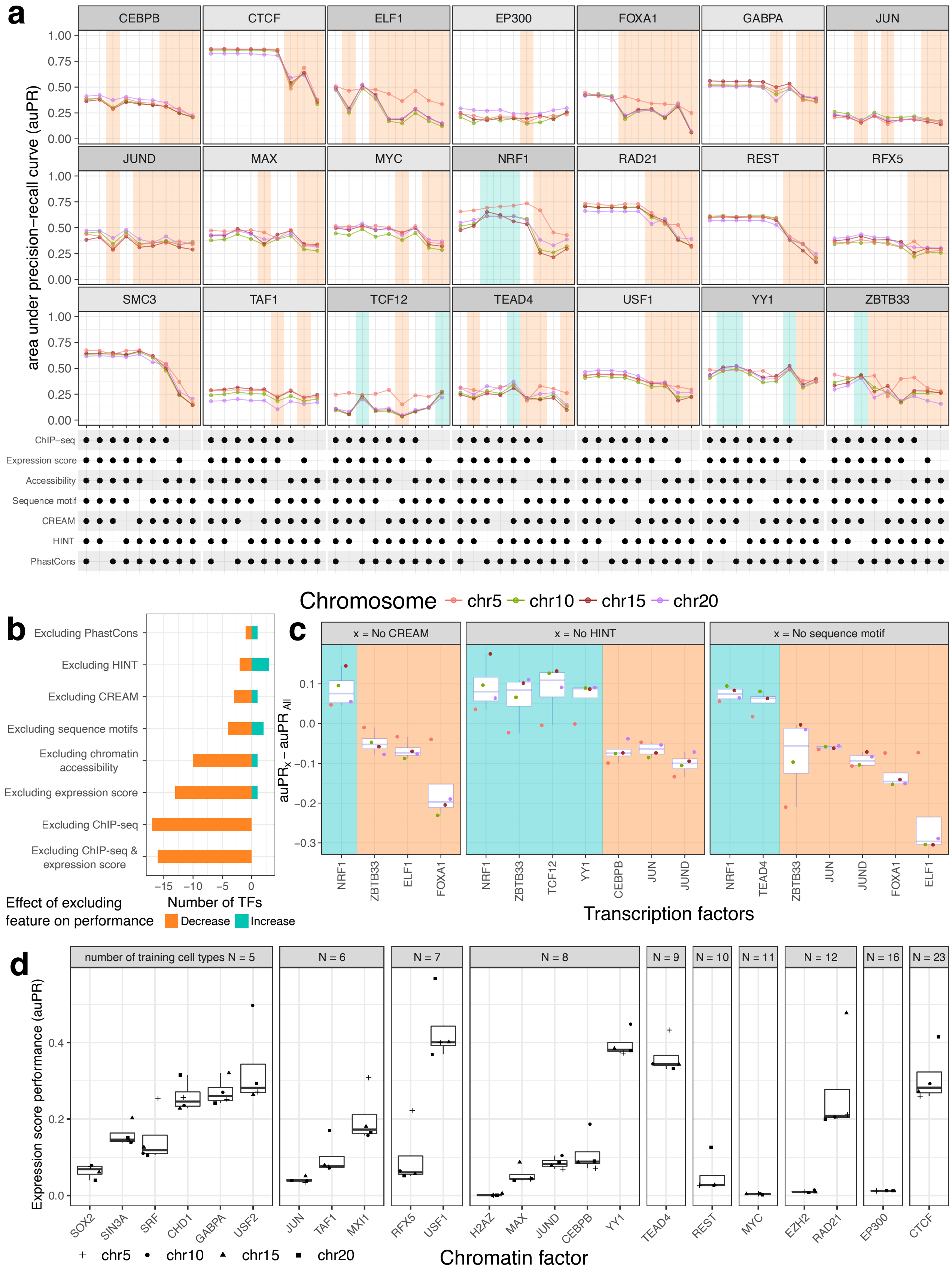

available total 23 cell types. The number of training cell types correlated weakly with auPR from

expression score alone (Pearson correlation $r = 0.1$). This may have been caused by the high similarity of CTCF binding across most cell types.

Inclusion of some features have opposite effects on prediction of different chromatin factors

Beyond the most important features—chromatin accessibility, ChIP-seq, and expression score—excluding other features rarely substantively decreased prediction performance (Fig. S4b–c). When we excluded sequence motifs, auPR decreased substantively for ZBTB33, JUN, JUND, FOXA1, and ELF1. Excluding HINT footprints decreased auPR substantively only for CEBPB, JUN, and JUND. Excluding CREAM clusters of chromatin accessibility peaks decreased auPR substantively only for ZBTB33, ELF1, and FOXA1.

Removing certain input features actually improved prediction for some chromatin factors (Fig. S4b–c). Associations that differed between training cell types and validation cell types suggested that these input features generalize poorly. For example, CREAM clusters' overlap with NRF1 ChIP-seq peaks was not consistent among GM12878 (7.52%), HeLa-S3 (31.8%), and HepG2 (25.78%). This represented a significant variation among these cell types (ANOVA; $p = 1.9 \times 10^{-4}$).

While most TF footprints (95.96%) overlapped NRF1 peaks, TF footprints constituted only a small fraction of NRF1 peaks (0.73%). NRF1 peaks overlapped a smalls proportion of TF footprints in training cell types GM12878 (1.14%) and HeLa-S3 (0.59%), but significantly greater than the 0.45% overlap in HepG2 (Welch t-test; $p = 0.007$). In HepG2, 7.28% of YY1 peaks overlap TF footprints while in the training cell type GM12878, the overlap is only 1.22% (Welch t-test; $p = 5 \times 10^{-5}$) and in the other training cell type HCT-116 the overlap is much higher (17.92%; Welch t-test; $p = 5 \times 10^{-6}$). Overlap of ZBTB33 peaks with TF footprints is much smaller in HepG2 (0.49%) compared to training cell types GM12878 (2.32%) and HCT-116 (5.27%; Welch t-test; $p = 6 \times 10^{-4}$). Features with varying and cell-specific association with chromatin factor binding complicate convergence of the multi-layer perceptron and may result in overfitting. As a result, the multi-layer perceptron achieved a higher performance on some chromatin factors when we ablated those features.

Association of clusters of regulatory elements and chromatin factor footprints with chromatin factor binding varies among cell types. Using a CREAM feature substantively improved performance in 3/21 chromatin factors and using a HINT feature substantively improved performance in 3/21 chromatin factors (Fig. S4b–c). In contrast, including CREAM substantively decreased performance for 1 case and including HINT for 4 cases. When we repeat this experiment by using different training and validation cell types, clusters of regulatory elements and TF footprints result in increase or decrease in performance of different chromatin factors, while they barely result in an increase in auPR above 0.05. Because of the limited upside and apparent downside, we didn't use these two cell-type–specific features for our final model.

TFs and their targets regulate similar biological pathways

Gene set enrichment analysis of chromatin factor targets

To calculate the expression score, we investigate correlation of chromatin factor binding at each genomic bin with expression of 5,000 genes across the genome (Methods). This brings us to our hypothesis that genes whose expression is perturbed with binding of a chromatin factor regulate the same biological processes as the chromatin factor. To understand biological implications of transcriptome perturbation in response to chromatin factor binding, we measured how frequently each gene's expression associated with binding of each chromatin factor. We hypothesized that if expression of a gene consistently correlates with binding of a chromatin factor, it is a potential target of that chromatin factor. Similarly, if the expression of a gene negatively correlates with binding of a chromatin factor, cellular machinery upregulated by that chromatin factor might cause net suppression of that gene's

expression.

To identify such genes, for each chromatin factor, we ranked genes by subtracting the number of genomic bins they are positively correlated with from the number of genomic bins they are negatively correlated. We call this difference the *association delta*. For each chromatin factor, we identified the 5,000 genes with the highest variance in expression among cells with matched RNA-seq and ChIP-seq data (Figure 1a). We measured correlation of expression of each of the 5,000 genes with chromatin factor binding at every 100 bp genomic window in 4 chromosomes (chr5, chr10, chr15, and chr20). This approach identified genes that have consistent positive or negative association with chromatin factor binding (Fig. S5a). We considered these genes as potential targets of each chromatin factor, and used the GSEA tool [62] to identify pathways with significant enrichment in either direction (Fig. S5a.) Only the rank of association delta affects these results, and we presumed that there would be little difference in using all chromosomes instead of just 4. The 4-chromosome analysis for JUND had no significant rank difference from an analysis of chromosome 10 alone (Wilcoxon rank sum test $p = 0.3$). We only investigated GO terms annotated to a minimum of 10 and a maximum of 500 out of a total of 17,106 GO-annotated genes.

We identified 1,681 GO terms with significant enrichment (GSEA $p < 0.001$) among potential targets of at least one of the 113 chromatin factors we investigated (Fig. S5b). Only 63 of these 113 chromatin factors had matched ChIP-seq and RNA-seq in at least 5 of the training cell types and one of the validation cell types we used for learning from the transcriptome. Each chromatin factor had potential targets with significant enrichment in a mean of 92 terms (median 76; Fig. S5c). Each of the 1,681 terms had significant enrichment in potential targets of a mean of 6 chromatin factors (median 2; Fig. S5d). Furthermore, 300 of these GO terms had significant enrichment in potential targets of at least 10 chromatin factors.

To identify chromatin factors involved in similar biological processes, we searched for enrichment of any of the 1,681 GO terms in 113 chromatin factors. This analysis relied on the GSEA enrichment score as a normalized test statistic. We examined the pairwise correlation between the vector of enrichment scores for each pair of chromatin factors. These pairwise correlations constitute a symmetric correlation matrix. We hypothesized that chromatin factors with high correlation are involved in similar biological processes.

To identify groups of chromatin factors involved in similar biological processes, we performed hierarchical clustering on the correlation matrix of enrichment of targets of each chromatin factor in various biological processes. We sought to identify clusters of chromatin factors, and the best number of clusters between 2 and 10, inclusive. As a control, we generated a correlation matrix of same dimensions from a matrix of random Gaussian values (Methods). For each matrix we repeatedly generated random subsamples and clustered them. For each subsample, we found the set of pairs of chromatin factors with the same cluster membership. For couples of these subsamples, we identified the Jaccard index between these sets as a measure of cluster stability [53] (Methods). We then compared the increase or decrease in Jaccard indices from each number of clusters to the number of clusters one larger.

The smallest number of clusters with an increase in Jaccard index only for the correlation matrix was 6 (Fig. S5e–f). We assigned names to these clusters based on their enriched biological pathways. We then examined the chromatin factors included in those clusters. The Neural cluster (Fig. S5g) includes ASCL1 [63], HSF1 [68], GATA2 [67], and PPARγ [69]. These chromatin factors play important roles in the development of the nervous system and are implicated in neurological disorders [63, 67, 68, 69]. The top 5 GO terms enriched in the potential targets of these chromatin factors are all related to nervous system development and function (Fig. S5g). The downregulated pathways of the Motility cluster (Fig. S5h) relate to cytoskeletal organization. The included chromatin factors, CTBP1 [73], KDM5B [74], MEF2A [75], and STAT1 [76], all play a role in the epithelial-to-mesenchymal transition, which involves re-organization of the cytoskeleton. Similarly, we found that for other clusters, specific upregulated or downregulated pathways of cluster's targets are also regulated by many of the cluster's chromatin factors (Fig. S5i–l; Table S1).

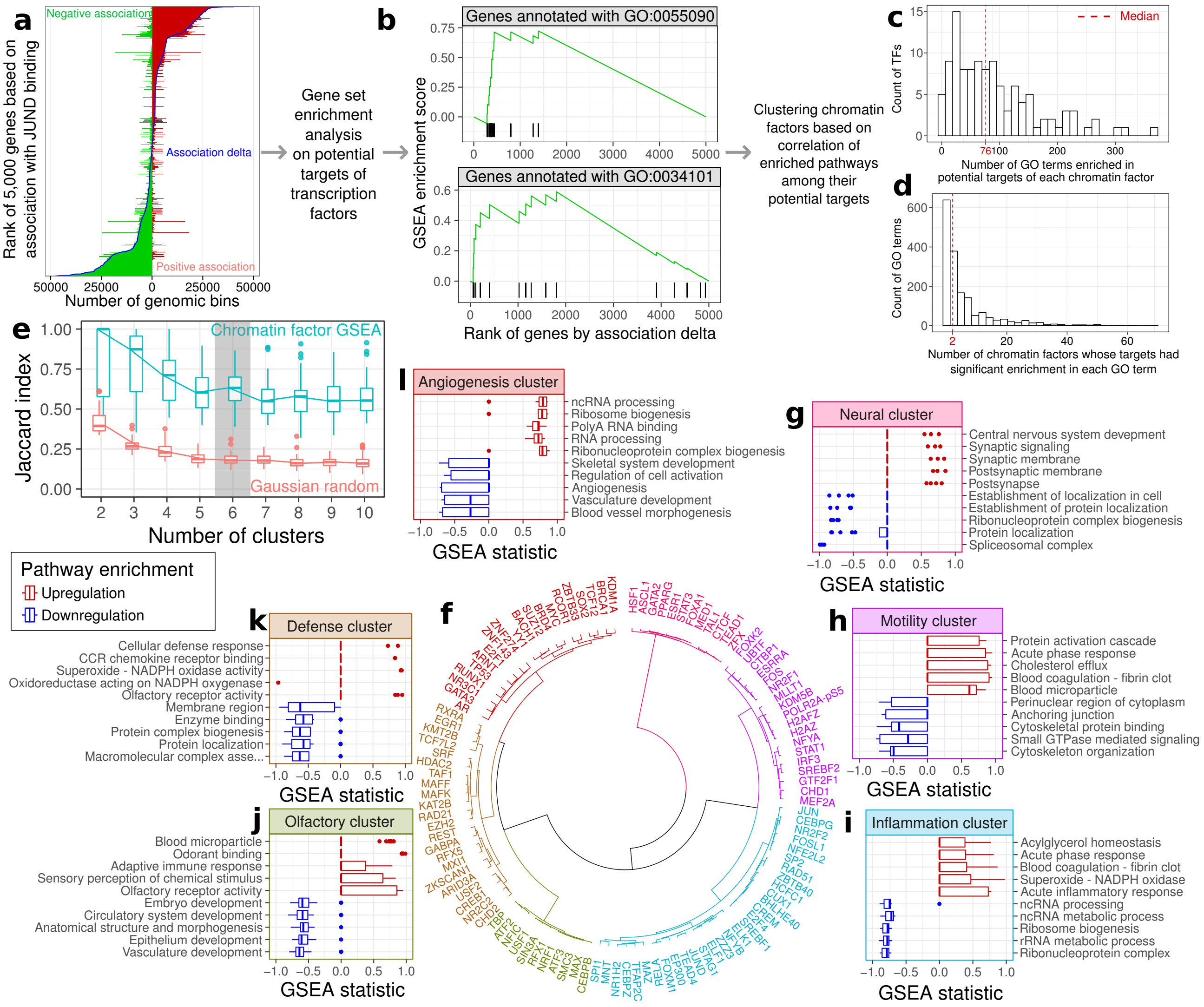

Fig. S5: **Top biological pathways regulated by potential targets of chromatin factor clusters.** Each gene may have both positive and negative correlation with chromatin factor binding at some genomic bins. For each chromatin factor, we ranked 5,000 genes by an association delta that summarizes how many genomic bins associated with binding. The association delta takes the number of bins that positively associated with a gene's expression and subtracts the number of bins that negatively associated. **(a)** The association ranking process for JUND binding. Double-ended bar plot for each of the 5,000 genes, with positive (red) and negative (green) association. Superimposed blue curve: association delta for each gene. **(b)** Gene Set Enrichment Analysis (GSEA) [62] identified pathways with significant enrichment in potential targets of each chromatin factor. Vertical black bars: rank of association delta for genes annotated with each Gene Ontology (GO) term. Green line: GSEA enrichment score. **(c)** Histogram showing how many of 1,681 GO terms are enriched in potential targets of each chromatin factor. **(d)** Histogram showing how many of 63 chromatin factors have potential targets with enrichment in each GO term. **(e)** Boxplot of cluster stability, as measured by Jaccard index, between clusters found in both the subsampled correlation matrix of chromatin factors by GSEA (turquoise) and a subsampled random Gaussian matrix of the same dimensions (red). Grey background: the smallest number of clusters where GSEA matrix cluster stability increased but that of the random Gaussian matrix did not. **(f)** Dendrogram of 6 clusters identified in the correlation matrix. We defined 6 clusters based on correlation of enrichment in 1,681 GO terms. **(g–l)** Boxplots of GSEA statistic for the top 5 pathways enriched in genes positively (red) and negatively (blue) correlated with chromatin factor binding for each cluster.

Chromatin factor cluster	Upregulated pathways	Downregulated pathways	Chromatin factors in cluster with relevant biology
Neural	**Neural activity and development**	Protein biosynthesis	ASCL1 [63], CTCF [64], ESR1 [65], FOXA1 [66], GATA2 [67], HSF1 [68], PPARγ [69], STAT3 [70], TAL1 [71], TEAD1 [72]
Motility	Inflammation	**Cytoskeletal organization**	CTBP1 [73], KDM5B [74], MEF2A [75], STAT1 [76]
Inflammation	**Inflammation**	RNA biosynthesis	BHLHE40 [77], CEBPG [78], CUX1 [79], ELK1 [80], FOXM1 [81], JUN [82], JUND [83], RELA [84]
Olfactory	**Olfactory perception**	Vasculature, blood, and structural development	NFIC [85], ATF2 [86], ATF3 [87], SIN3A [88], CEBPB [89], RFX1 [90]
Defense	**Cell defense and chemokine signaling**	Protein biogenesis and localization	ARID3A [91], CREB1 [92], EGR1 [93], KAT2B [94], KMT2B [95], MAFF [96], RFX5 [97], RXRA [98], SRF [99]
Angiogenesis	RNA biosynthesis	**Angiogenesis and vasculature**	AR [100], ARNT [101], BACH1 [102], BRCA1 [103], BRD4 [104], E2F1 [105], GATA3 [106], KDM1A [107], MYC [108], RUNX1 [109], TP53 [110]

Table S1: **Many chromatin factors within each biological function cluster are involved in the same pathways as their potential target genes.** We summarized each cluster of chromatin factors according to top over-represented GO terms in the first 3 columns. Chromatin factors in the 4th column are involved in the same biological mechanism as the bold pathways mentioned in 2nd or 3rd column.

Avocado comparison

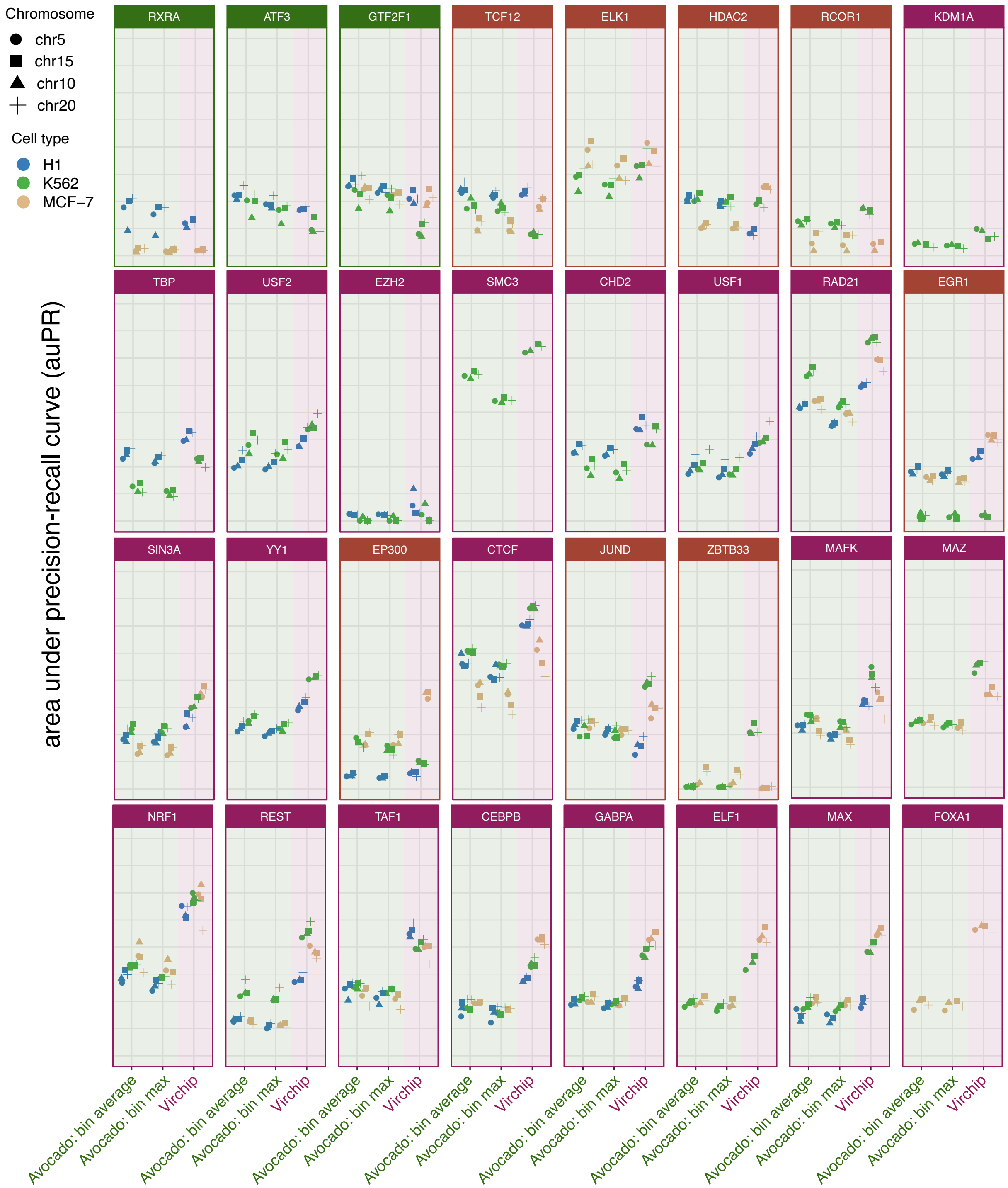

Fig. S6: **Area under the Precision-recall curve (auPR) of Virtual ChIP-seq and Avocado predictions on 3 cell types.** Left green label: Mean Avocado imputations over 8 25 bp bins; middle green label: maximum Avocado imputations over 8 25 bp bins; right purple label: Virtual ChIP-seq predictions in 200 bp bins. We tested the predictions on 3 cell types: H1-hESC (blue), K562 (green), and MCF-7 (khaki). We examined chr5 (circle), chr10 (triangle), chr15 (square), and chr20 (cross). Facets show 32 chromatin factors used for the comparison. Facet color shows whether the best performance in all 3 cell types came from Avocado (green) or Virtual ChIP-seq, or whether best performance was mixed between the two methods (brown).
