# Supplementary material for: Virtual ChIP-seq: predicting transcription factor binding by learning from the transcriptome
Source: Genome Biol. 2022 Jun 10;23:126. doi: 10.1186/s13059-022-02690-2 (PMC9185870; doi:10.1186/s13059-022-02690-2)
Supplement: Supplementary file 3 — Additional file 3 Review history. [file 13059_2022_2690_MOESM3_ESM.docx]

This manuscript was previously reviewed at another journal. We have seen the reviews, but are not able to publish them. The revised manuscript was reviewed again by one of the original reviewers, and their review is here:

The authors have thoroughly addressed my previous concerns. I especially appreciate the inclusion of an additional benchmark against the Avocado method.

Minor essential comments:

1. From the current typeset manuscript, it seems that the Methods section might be intended to be provided as a supplement, because it is placed after Figures and References. I would strongly suggest to place the Methods within the main manuscript.

2. The installation of virchip via conda following the instructions in the github repository does not work, currently. I assume, the reason could be that the source url in the recipe's meta.yaml still points to a bitbucket repository.

3. I did not find the Supplementary Table listing "44 sequence motif features from Epigram and DNase-seq peaks provided by the authors". Supplementary Table 10 (mentioned in the manuscript) is about the class imbalance in DREAM and Cistrome data sets, Supplementary Table 12 (mentioned in the authors' response) lists training and validation cell types for the ablation study.

Minor discretionary comments:

4. In Figure 3c, the symbols displayed in the legend do not perfectly match those in the panels. Specifically, the triangles in the panels point upwards for Virtual ChIP-seq and Catchitt, whereas in the legend the triangle of Virtual ChIP-seq points downwards.

5. In Supp. Fig. 6, the points within the panels are overlapping substantially, and the spacing between the columns for the three methods within a panel is too low to clearly attribute points to either method in some cases. I would suggest to use the complete page for Supp. Fig. 6 and to increase the width of the individual panels.
